# Basal autophagy is required for promoting dendritic terminal branching in *Drosophila* sensory neurons

**DOI:** 10.1371/journal.pone.0206743

**Published:** 2018-11-05

**Authors:** Sarah G. Clark, Lacey L. Graybeal, Shatabdi Bhattacharjee, Caroline Thomas, Surajit Bhattacharya, Daniel N. Cox

**Affiliations:** 1 Neuroscience Institute, Georgia State University, Atlanta, Georgia, United States of America; 2 School of Systems Biology, Krasnow Institute for Advanced Study, George Mason University, Fairfax, Virginia, United States of America; Children's Hospital of Pittsburgh, University of Pittsburgh Medical Center, UNITED STATES

## Abstract

Dendrites function as the primary sites for synaptic input and integration with impairments in dendritic arborization being associated with dysfunctional neuronal circuitry. Post-mitotic neurons require high levels of basal autophagy to clear cytotoxic materials and autophagic dysfunction under native or cellular stress conditions has been linked to neuronal cell death as well as axo-dendritic degeneration. However, relatively little is known regarding the developmental role of basal autophagy in directing aspects of dendritic arborization or the mechanisms by which the autophagic machinery may be transcriptionally regulated to promote dendritic diversification. We demonstrate that autophagy-related (*Atg*) genes are positively regulated by the homeodomain transcription factor Cut, and that basal autophagy functions as a downstream effector pathway for Cut-mediated dendritic terminal branching in *Drosophila* multidendritic (md) sensory neurons. Further, loss of function analyses implicate *Atg* genes in promoting cell type-specific dendritic arborization and terminal branching, while gain of function studies suggest that excessive autophagy leads to dramatic reductions in dendritic complexity. We demonstrate that the Atg1 initiator kinase interacts with the dual leucine zipper kinase (DLK) pathway by negatively regulating the E3 ubiquitin ligase Highwire and positively regulating the MAPKKK Wallenda. Finally, autophagic induction partially rescues dendritic atrophy defects observed in a model of polyglutamine toxicity. Collectively, these studies implicate transcriptional control of basal autophagy in directing dendritic terminal branching and demonstrate the importance of homeostatic control of autophagic levels for dendritic arbor complexity under native or cellular stress conditions.

## Introduction

Neurons exhibit a vast array of morphological architectures due in part to their distinct and elaborate patterns of dendritic arborization. Dendritic arbor diversity across neuronal subtypes plays a pivotal role in regulating synaptic and sensory integration, functional connectivity, electrotonic properties and neuronal computation [1]. Therefore, it is important for both intrinsic and extrinsic cues to coordinately trigger the precise molecular mechanisms needed to specify, maintain, and modulate cell type-specific dendritic architecture and thereby promote proper neuronal function. Such cues include molecules involved in cell adhesion, the secretory pathway, synaptic signaling, cytoskeletal regulation, and transcriptional regulation [1–3].

In addition to these cellular processes, recent studies demonstrate that the autophagy pathway is one mechanism involved in maintaining neuronal morphology that is evolutionarily conserved across species including *C*. *elegans*, *D*. *melanogaster* and mammals [4]. Macroautophagy (referred to hereafter simply as autophagy or basal autophagy) is the cell-mediated clearance and recycling of ubiquitinated cytosolic components, such as damaged organelles and protein aggregates, which occurs at basal levels as a housekeeping function [4,5]. Autophagy has been demonstrated to play a wide variety of mechanistic roles in regulating cellular homeostasis, as well as remodeling in terminally differentiated cells of both invertebrates and vertebrates [4–8]. During autophagy, sequestered cytoplasmic materials are engulfed by vesicles termed autophagosomes. These later fuse with endolysosomes to degrade vesicular contents into reusable molecules and sources of energy, which provide nutrients during periods of starvation or cellular stress [9,10]. The autophagy process consists of several phases, each involving a different group of proteins encoded by the evolutionarily conserved *Atg* genes [4,11]. These phases include autophagic induction, cargo recognition and packaging, Atg protein cycling, vesicle nucleation, vesicle completion, and fusion with the lysosome [12].

Post-mitotic neurons are known to require high levels of basal autophagy for cellular homeostasis in terms of clearing misfolded proteins and damaged organelles [4]. Autophagic dysfunction—both at basal levels and during periods of cellular stress—has been correlated to various types of neurodegeneration including neuronal cell death, axo-dendritic degeneration, and aberrant synapse development [13–16]. This suggests that autophagy has a neuroprotective function [4,17,18]. Moreover, disruption of *Atg* genes and autophagic function has been shown to lead to the accumulation of ubiquitin-positive and other abnormal protein aggregates known to contribute to a variety of neurodegenerative disease states including Parkinson’s and Huntington’s [4,17–19].

Despite the importance of the autophagy pathway in neuronal function, the transcriptional mechanisms controlling cell type-specific expression of *Atg* genes and the developmental role of basal autophagy in promoting dendritic arbor diversity both remain largely unknown. Furthermore, while significant evidence has emerged that complex transcriptional regulatory programs function to generate the array of neuronal dendritic architectures, much remains to be discovered regarding the downstream cellular and molecular mechanisms through which these transcriptional codes are implemented to drive dendritic diversification [3,20]. *Drosophila* has proven a powerful model for investigating autophagy due to the evolutionary conservation of the core machinery involved in the autophagic process [7,11]. Moreover, *Drosophila* multidendritic (md) sensory neurons have served as a robust system for characterizing dendrite morphogenesis [21]. These sensory neurons lie just beneath the barrier epidermis and are subdivided into four distinct morphological classes ranging from the relatively simple Class I (C-I) neurons that display selective dendritic field coverage to the more complex Class III (C-III) and Class IV (C-IV) neurons that display dendritic space-filling and tiling properties. These properties facilitate dissection of cellular and molecular underpinnings driving cell type-specific dendritic diversification and homeostasis [21,22].

Here we functionally connect transcriptional regulation to autophagy in directing cell type-specific dendritic arborization in *Drosophila* md sensory neuron subtypes. We demonstrate that the homeodomain transcription factor Cut positively regulates the expression of *Atg* genes linked to autophagic induction, Atg protein cycling, and vesicle completion and that basal autophagy functions as a downstream effector of Cut-mediated dendritic terminal branching. Genetic analyses reveal that insufficient or excessive autophagic activity leads to defects in dendritic arborization and higher order complexity indicative of a homeostatic role for autophagy in directing cell type-specific features contributing to dendritic diversification. Genetic interaction studies identify a regulatory relationship between autophagy and the DLK pathway. Finally, we demonstrate that under conditions of cellular stress, autophagic induction can partially rescue dendritic atrophy phenotypes exhibited in a model of polyglutamine toxicity, providing a link between upregulation of autophagy and mitigation of dendritic neurodegenerative-like defects.

## Materials and methods

### *Drosophila* genetics

*Drosophila* stocks were maintained at 25°C on standard molasses-cornmeal agar. The following strains were obtained from Bloomington *Drosophila* Stock Center: UAS-RNAi lines (*cut*^*HMS00924*^ [23]*; Atg1*^*JF02273*^*; Atg1*^*GL00047*^*/TM3*, *Sb*^*1*^*; Atg2*^*HMS01198*^*; Atg2*^*JF02786*^*; Atg5*^*HMS01244*^*; Atg5*^*JF02703*^*; Atg8a*^*JF02895*^*/TM3*,*Sb*^*1*^*; Atg8a*^*HMS01328*^*; Atg18*^*JF02898*^*; Atg18*^*HMS01193*^*); GAL4*^*477*^,*UAS-mCD8*::*GFP; UAS-Atg1*^*6A*^*; UAS-Atg1*^*6B*^*; UAS-GFP-hiw*^*A*^; *UAS-MJD-78Q; UAS-eGFP-Atg5; UAS-Atg8a*.*GFP; UAS-wndK188A*. Additional strains from other sources included the pan-md reporter strain *GAL4*^*21-7*^,*UAS-mCD8*::*GFP* [24]; class I md reporter strain *GAL4*^*221*^,*UAS-mCD8*::*GFP* [25]; class III md neuron reporter strains *GAL4*^*19-12*^,*UAS-hCD4*::*tdGFP* [26]*; nompC-GAL4*,*UAS-mCD8*::*GFP* [27]; and *ppk-GAL4*,*UAS-mCD8*::*GFP*, *ppk-GAL80* [23]; class IV md neuron reporter strains *GAL4*^*477*^,*UAS-mCD8*::*GFP; ppk1*.*9-GAL4*,*UAS-mCD8*::*GFP* [28–30]; and *ppk*::*hCD4*::*tdTomato* (donated by Dr. Yuh-Nung Jan) [31]*; UAS-cut; UAS-Atg1*^*K38Q*^ (donated by Dr. Thomas Neufeld, University of Minnesota) [32], *UAS-Hiw*^*ΔRING*^ and *UAS-wnd*.*C* (donated by Dr. Catherine Collins, University of Michigan) [33,34]. A minimum of two independent gene-specific *UAS-RNAi* (IR) lines were used for each *Atg* gene to control for any potential off-target effects and a representative IR line for each *Atg* gene is depicted in the figures. Detailed genotypes for each figure are reported in S1 Table.

### Cell isolation and microarray expression profiling

Class-specific isolation and purification of md neurons was performed as previously described [30,35,36]. For C-I, III, and IV profiling, neurons were extracted from *GAL4*^*2-21*^,*UAS-mCD8*::*GFP* (C-I) [36], *ppk-GAL4*,*UAS-mCD8*::*GFP*, *ppk-GAL80* (C-III) [37], and *ppk1*.*9-GAL4*,*UAS-mCD8*::*GFP* (C-IV) [30] age-matched third instar larvae, respectively. For comparative expression profiling between WT C-I neurons and those ectopically overexpressing Cut, neuronal isolations were taken from third instar larvae expressing *UAS-mCD8*::*GFP* under the control of *GAL4*^*2-21*^ in the presence or absence of *UAS-cut* [36]. mRNA isolation, amplification, labelling, hybridization, and microarray analyses was then performed by Miltenyi Biotec from the class-specific neuronal isolations as previously described [30,35–37]. Microarray data, including metadata, raw data and quantile normalized datasets have been deposited into the Gene Expression Omnibus (GEO) under the following accession numbers: GSE46154 (WT C-I and C-IV neurons) [30]; GSE69353 (WT C-III neurons) [37]; and GSE83938 (WT C-I and C-I neurons ectopically overexpressing Cut) [36]. All microarrays were performed on identical Agilent whole *D*. *melanogaster* genome oligo microarrays (4x44K) and statistical analyses of microarray data were performed as previously described [38]. Raw microarray data files obtained from the Agilent microarrays for each md neuron class were read into GeneSpring GX software in which the data was quantile normalized and only those gene probes which were flagged positive and significantly expressed above background were selected for further analyses. GeneSpring software was used to calculate mean fold change gene expression where a given gene/isoform was represented by multiple probe IDs on the microarray.

### Immunofluorescent labeling

Dissection and immunofluorescent labeling of third instar larval filets was performed as previously described [39]. Primary antibodies used in this study include: rabbit anti-GABARAP/Atg8a (1:200) (Abcam), rat anti-Atg8a (1:320) (donated by Dr. Gábor Juhász, Eötvös Loránd University) [40], rabbit anti-WndA1 (1:300) (donated by Dr. Catherine Collins, University of Michigan) [34], Dylight AffiniPure Goat anti-horseradish peroxidase (HRP) 488 and 549 conjugated (1:200), AlexaFluor Goat anti-HRP 647 conjugated (1:200). Secondary antibodies used include: donkey anti-rat (1:1600) (Jackson Immunoresearch) and donkey anti-rabbit (1:200) (Life Technologies). Filets were imaged on either a Nikon C1 Plus confocal microscope or a Zeiss LSM780 confocal microscope and fluorescence intensities quantified using the Measure–mean gray value function in ImageJ [41] and were normalized to area to control for differences in md neuron subclass cell body size. Identical confocal settings for laser intensity and other image capture parameters were applied for comparisons of control vs. experimental samples.

### Live imaging confocal microscopy, neuronal reconstruction, and morphometric data analyses

Live neuronal imaging was performed as previously described [23,30]. We focused on the dorsal cluster of md neurons including C-I ddaE neurons; C-III ddaF and ddaA neurons; and C-IV ddaC neurons as morphological representatives of these md neuron subclasses. Dendritic morphology was quantified as previously described [30]. Briefly, maximum intensity projections of confocal Z-stacks were exported as a jpeg or TIFF. Once exported, images were manually curated to eliminate non-specific auto-fluorescent spots (such as the larval denticle belts) using a custom designed program, *Flyboys* (freely available upon request). For total dendritic length measurements, images were processed and skeletonized in ImageJ [30,41]. Quantitative neuromorphometric information was extracted and compiled using custom Python algorithms. The custom Python scripts were used to compile the output data from the Analyze Skeleton ImageJ plugin and the compiled output data was imported into Excel (Microsoft). For total dendritic branches and number of terminal branches, images were reconstructed using NeuronStudio [42]. Branch number and order were then extracted using the centripetal branch labeling function and output data was compiled in Excel.

### qRT-PCR

qRT-PCR analysis of WT and *cut-IR* expressing neurons was done in quadruplicates as previously described [23]. Briefly, *UAS-mCD8*::*GFP* expressing C-IV md neurons were isolated using superparamagnetic beads (Dynabeads MyOne Streptavidin T1, Invitrogen) that were coupled to biotinylated anti-CD8a antibody (eBioscience). RNA was then isolated from these cells using the miRCURY RNA Isolation Kit (Exiqon) and qRT-PCR was performed using the following pre-validated QuantiTect Primer Assays: *Atg1* (QT00963536), *Atg2* (QT00956963), *Atg5* (QT00499723), *Atg8a* (QT00919695) and *Atg18* (QT00960337). Expression data was normalized to *RpL32* (QT00980007).

### Statistical analysis and data availability

Statistical analyses of neuromorphometric data and data plotting were performed using GraphPad Prism 7. Error bars reported in the study represent SEM. Statistical analyses were performed using either two-tailed unpaired t-test with Welch’s correction or one-way ANOVA using Dunnett’s multiple comparisons test when data sets were normally distributed as determined by the Shapiro-Wilk normality test. When data was not normally distributed, appropriate non-parametric tests were used (see figure legends for specific tests used in each case). Significance scores indicated on graphs are (* = p≤0.05, ** = p≤0.01, *** = p≤0.001). Detailed information on statistical analyses for each figure is reported in S2 Table. All newly reported genotypes presented here are available upon request. The microarray data is publicly available via the following GEO accession numbers: GSE46154, GSE69353, and GSE83938.

## Results

### Cut positively regulates the expression of *Atg* genes

The homeodomain transcription factor Cut has been shown to promote dendritic diversification in morphologically complex C-III and C-IV md neurons via regulation of a variety of cellular processes including the secretory pathway and the cytoskeleton [3,23,36,43]. Previous studies have demonstrated that Cut protein is differentially expressed in md neuron subclasses with the highest levels in C-III neurons, followed by moderate and low levels in C-IV and C-II md neurons, respectively [25]. The levels of Cut expression are diversified in md neuron subclasses via the transcriptional regulators Scalloped and Vestigial [44]. In a recent study, we reported on comparative neurogenomic profiling of Cut-mediated transcriptional targets implicating a large number of differentially expressed genes and cellular processes that are positively regulated by Cut [36]. For these analyses, we capitalized on the observation that Cut is not normally detectable in C-I md neurons and therefore ectopic expression of Cut in these neurons can be used to examine Cut-mediated gene expression relative to control C-I neurons. This neurogenomic strategy also eliminates potential experimental confounds that may result from Cut overexpression in md neuron subclasses that normally express Cut [36].

**Fig 1:**
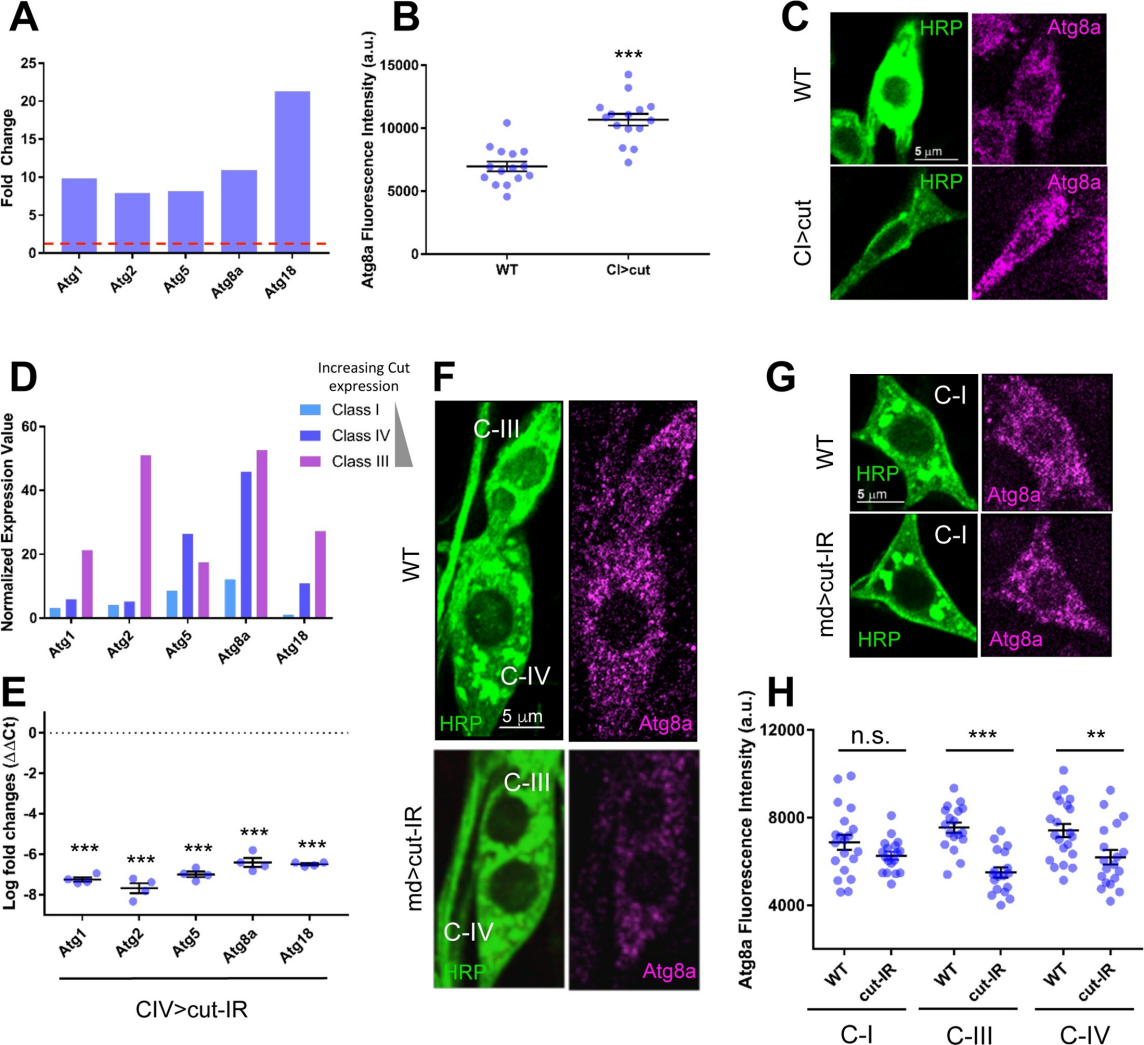
Cut positively regulates the expression of *Atg* genes. (A) Microarray analyses reveal that ectopic expression of Cut in C-I md neurons results in upregulation of select *Atg* genes. Data is presented as average fold change in expression from wild-type (WT) control C-I md neurons (red dashed line). (B) C-I md neurons ectopically expressing Cut (CI>cut) show a significant (p<0.0001) increase in Atg8a protein expression as compared to WT. (C) Representative confocal images of C-I md neuron cell bodies from third instar larval WT control and C-I driven *cut* overexpression (CI>cut) genetic backgrounds. Third instar larval filets were stained with anti-Atg8a antibodies to mark autophagosomes and fluorescently-conjugated HRP to label md sensory neurons. (D) Normalized average expression values for *Atg* genes based on WT class-specific (C-I, C-III, C-IV) microarray analyses. Key includes graphic depicting increasing Cut protein expression levels by md neuron subclass. (E) qRT-PCR analyses reveal that knockdown of *cut* in C-IV neurons results in substantial reduction of mRNA levels for the selected *Atg* genes. (F,G) Representative confocal images of C-I (G), C-III, and C-IV (F) md neuron cell bodies from third instar larval WT control and pan-md driven *cut* RNAi knockdown (md>*cut-IR*) genetic backgrounds. Third instar larval filets were stained with anti-Atg8a antibodies to mark autophagosomes and fluorescently-conjugated HRP to label md sensory neurons. (H) Quantitative analyses of Atg8a fluorescence intensity normalized to area in C-I, C-III, and C-IV md neurons reveals significantly reduced expression in both C-IV and C-III neurons in *cut-IR* relative to WT controls. There is no significant change in Atg8a levels in C-I md neurons between *cut-IR* and WT controls. Statistics: two-tailed unpaired t-test with Welch’s correction (B,H) or one-way ANOVA using Dunnett’s multiple comparisons test (E) (** = p≤0.01, *** = p≤0.001). For detailed genotypes see S1 Table. For detailed statistics see S2 Table. a.u. = arbitrary units. Quantitative data is reported as mean ± SEM in all figures unless indicated otherwise.

Bioinformatic analyses of Cut-mediated differential gene expression identified a variety of intriguing cellular pathways including coordinated upregulation of numerous *Atg* genes. Relative to wild-type (WT) control C-I md neurons, Cut misexpressing C-I neurons exhibited significantly increased expression of the following *Atg* genes (Fig 1A): *Atg1*, encoding an autophagy-specific serine/threonine protein kinase involved in the induction of autophagosome biogenesis [32]; *Atg2*, encoding a vacuolar protein sorting (VPS)-associated 13 domain containing protein involved in Atg protein cycling between peripheral sites and the phagophore assembly site (PAS); *Atg5*, encoding an E3-like ubiquitin ligase component involved in autophagosome vesicle completion via lipidation of Atg8; *Atg8a*, encoding a ubiquitin-like protein involved in autophagosome vesicle completion; and *Atg18*, encoding a WD40 repeat domain phosphoinositide-interacting protein involved in protein cycling and autophagosome formation [12].

To validate the putative regulatory relationship between Cut and Atg proteins independent of the neurogenomic analyses, we performed immunofluorescence analyses to determine whether Cut can directly increase Atg protein expression. For these analyses we examined the expression of Atg8a based upon characterized and available antibodies. Relative to WT control C-I md neurons, we observed a significant increase in Atg8a expression levels in C-I neurons ectopically expressing Cut (p<0.0001) (Fig 1B and 1C).

As Cut protein is not normally detectable in C-I neurons, we next investigated whether patterns of *Atg* gene expression in C-III and C-IV md neurons displayed any relationship to Cut protein expression levels in these neurons. Based upon previously published WT C-I, C-III, and C-IV md neuron transcriptome expression profiling data [30,36,37], we discovered that the normalized mRNA expression values of the *Atg* genes identified above were largely correlated with differential Cut protein expression levels. With the exception of *Atg5*, we observed the highest levels of *Atg* gene expression in C-III neurons followed by C-IV neurons, which corresponds with previously reported Cut differential expression levels (Fig 1D) [25]. Between md neuron subclasses, *Atg* genes were relatively enriched in C-III and C-IV neurons compared to C-I neurons (Fig 1D). To further confirm the regulatory relationship between Cut and *Atg* genes we demonstrate that *cut* knockdown in C-IV neurons results in significant reductions in the mRNA expression levels of all five of these genes as measured by qRT-PCR (p<0.0001 for all five genes) (Fig 1E).

To complement the mRNA expression analyses, we performed RNAi-mediated knockdown of *cut* (*UAS-cut-IR*) using a pan-md *GAL4* driver and labeled third instar larval filets with anti-Atg8a. We focused our analyses on C-I, C-III, and C-IV md neurons based upon differential Cut protein expression levels and our neurogenomic analyses. In WT control neurons, Atg8a protein expression appeared in punctate cytosolic vesicular structures representing presumptive autophagosomes throughout the md neuron cell bodies (Fig 1F and 1G). Quantitative analysis of C-I, C-III, and C-IV neurons in the dorsal cluster of md neurons revealed significant reductions in Atg8a protein expression levels with *cut* knockdown in the C-III (p<0.0001) and C-IV (p = 0.0093) md neurons compared to WT controls, supporting a positive regulatory relationship between Cut and Atg8a (Fig 1F and 1H). In contrast, we observed no significant change in Atg8a protein expression upon *cut* knockdown in the C-I md neurons (p = 0.1203), which was predicted based upon the lack of detectable Cut protein expression in C-I neurons [25] (Fig 1G and 1H). Collectively, these findings indicate that Cut positively regulates components of the autophagic machinery.

### Basal autophagy functions as a downstream effector of Cut-mediated dendritic terminal branching

**Fig 2:**
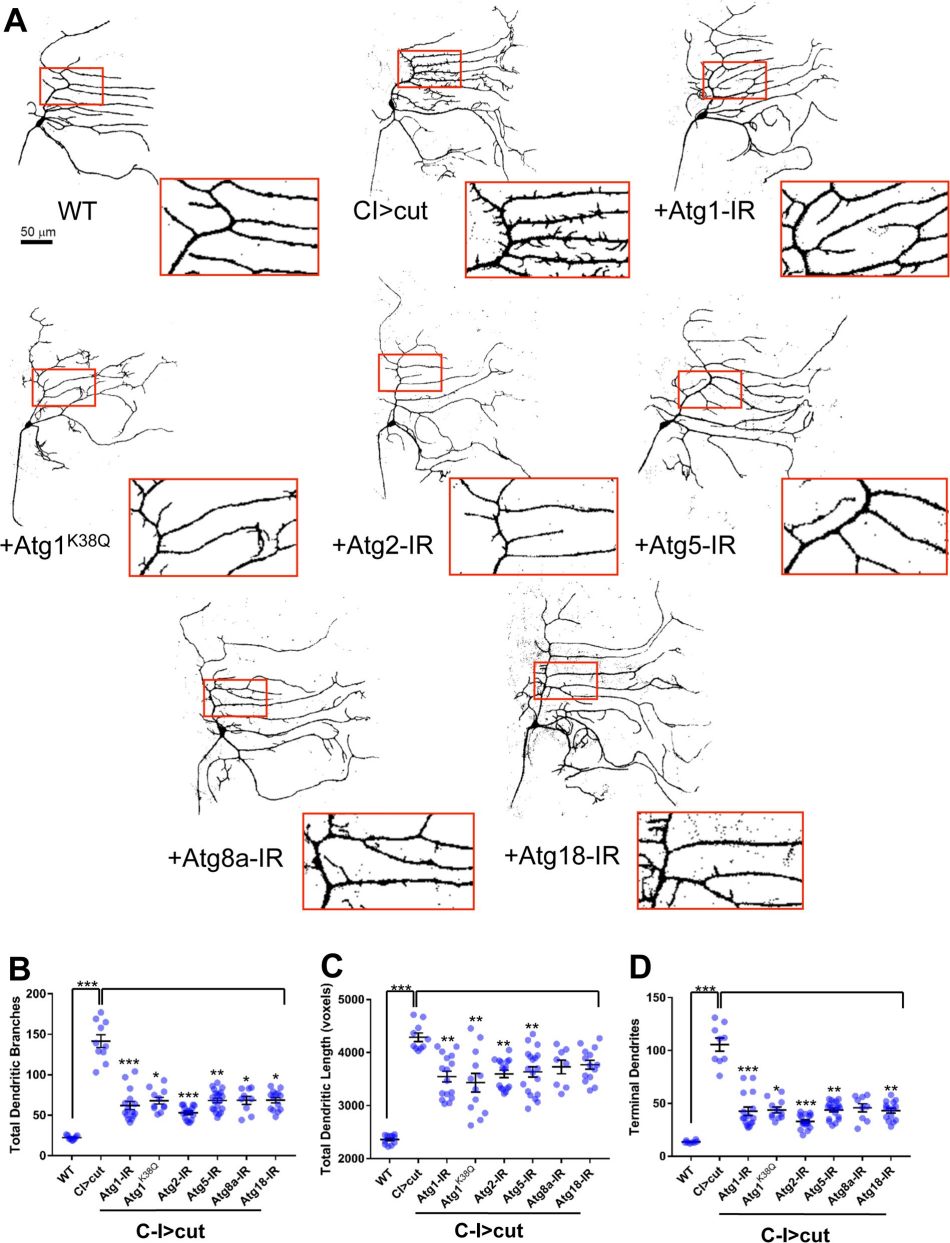
Autophagy is required for Cut-mediated dendritic terminal branching. (A) Representative confocal images of third instar larval C-I ddaE md neurons. Compared to WT, ectopic expression of Cut (CI>cut) dramatically increases dendritic branching complexity. Simultaneous misexpression of Cut and gene-specific *UAS-RNAi* transgenes for select *Atg* genes (*e*.*g*. *+Atg1-IR*) reveals suppression of Cut-mediated increases in dendritic growth and terminal branching compared to Cut misexpression alone. Insets present magnified view of corresponding dendritic regions between genotypes. (B-D) Quantitative neuromorphometric analyses of the total number of dendritic branches (B), total dendritic length in voxels (C) and number of terminal dendrites (D). Statistics: Kruskal-Wallis test using Dunn’s multiple comparisons test (* = p≤0.05, ** = p≤0.01, *** = p≤0.001. For detailed genotypes see S1 Table. For detailed statistics see S2 Table.

The regulatory relationship identified between Cut and *Atg* genes suggests that Cut may utilize the autophagy pathway to drive md neuron dendritic development and perhaps to regulate specific aspects of dendritic morphogenesis that contribute to md neuron subtype-specific dendritic diversification. If Cut requires these *Atg* genes, then we would predict that *Atg* gene disruption should result in a suppression of Cut-mediated dendritogenesis resulting from Cut ectopic expression in C-I md neurons. Relative to WT controls, ectopic expression of Cut in C-I md neurons (CI>cut) causes a dramatic increase in dendritic branching complexity characterized by extensive *de novo* formation of dendritic terminal filopodia-like branches emanating from the primary arbors (Fig 2A). Cut ectopic expression in C-I neurons results in significant increases in the total number of dendritic branches (Fig 2B), total dendritic length (Fig 2C), and number of terminal dendrites (Fig 2D). To test the hypothesis that *Atg* genes act as downstream effectors of Cut-mediated dendritogenesis in these neurons, C-I neurons ectopically expressing Cut were phenotypically compared to C-I neurons in which Cut was ectopically expressed with simultaneous expression of gene-specific RNAi for *Atg* genes identified as putative Cut targets. We have previously established that suppression of the Cut ectopic expression phenotype is not the result of GAL4 titration via co-expression of a neutral *UAS-mCD8*::*RFP* transgene [23], thus any morphological suppression effects are attributable to *Atg-IR* expression rather than GAL4 titration. Compared to C-I neurons ectopically expressing Cut alone, C-I neurons co-expressing Cut and *Atg* gene-specific RNAi transgenes (*Atg-IR*) displayed suppression of Cut-mediated *de novo* dendritic arborization, which was particularly notable with respect to Cut-induced short terminal dendritic filopodial branching (Fig 2A, compare insets). Quantitative neuromorphometric analyses support this observation as knockdown of *Atg* genes largely resulted in significant reductions in the total number of dendritic branches, total dendritic length, and number of terminal dendrites with a few exceptions (Fig 2B–2D, S2 Table). In most cases, there were significant concomitant reductions in the total number of branches and number of terminal dendrites with *Atg* gene knockdown (Fig 2B–2D) indicative of a requirement for autophagy in promoting the formation of Cut-induced *de novo* dendritic filopodial terminals. In addition to knockdown analyses, we drove expression of *UAS-Atg1*^*K38Q*^, a kinase dead transgene of *Atg1* [32], in C-I neurons ectopically expressing Cut. Expression of the kinase dead transgene competitively inhibits native Atg1 thereby impairing autophagic function through a reduction in kinase activity and autophagosome biogenesis [32]. Quantitative analyses of C-I neurons co-expressing Cut and *UAS-Atg1*^*K38Q*^ revealed significant reductions for all neuromorphometric parameters examined (Fig 2B–2D). These findings suggest that Cut-mediated dendritic terminal branching requires the basal autophagy pathway.

**Fig 3:**
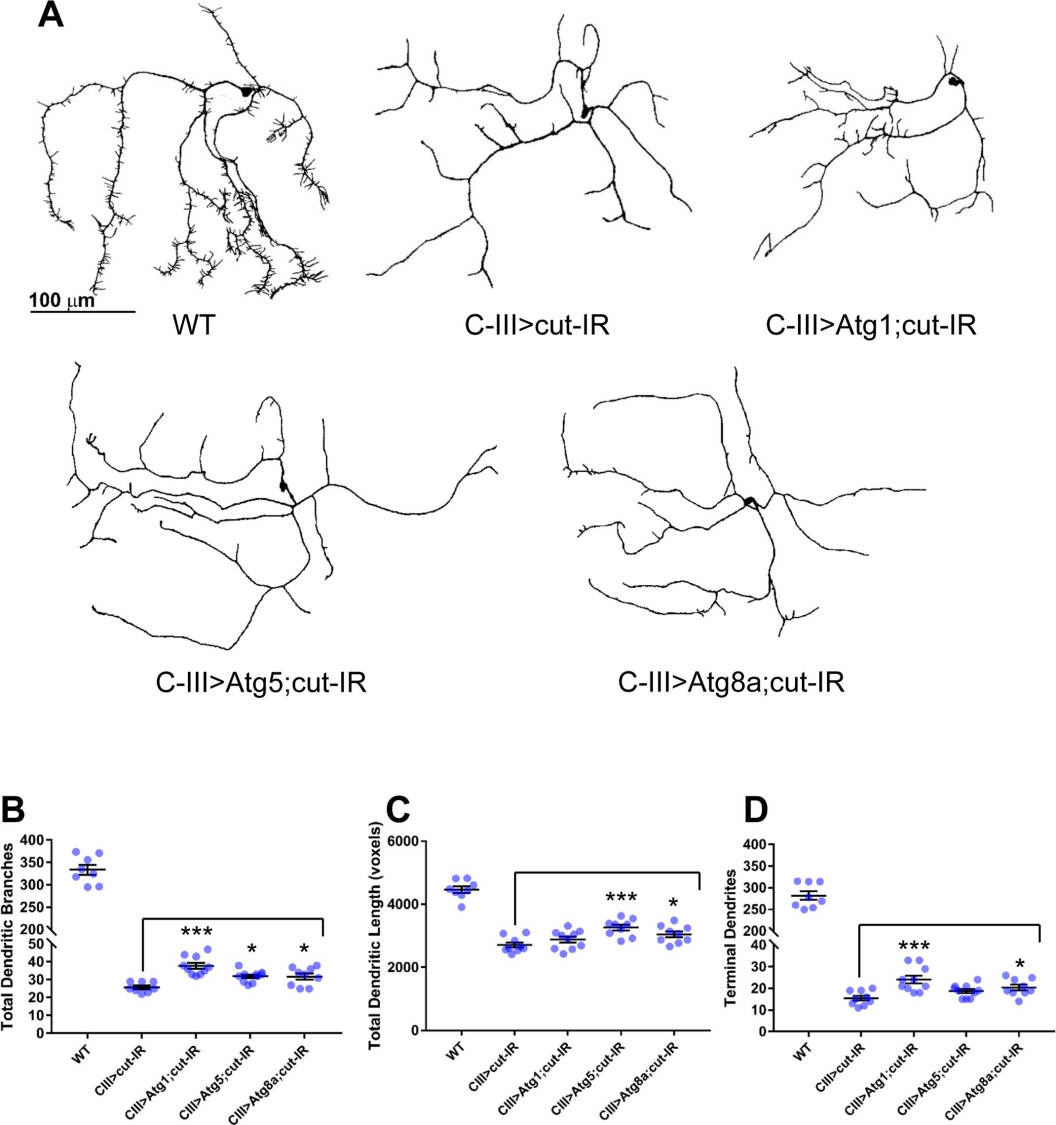
Autophagy partially rescues dendritic complexity deficits caused by *cut* knockdown in C-III md neurons. (A) Representative confocal images of third instar larval C-III ddaA md neurons. Compared to WT, expression of *cut-IR* results in severe loss of terminal dendritic filopodia-like branching. Simultaneous expression of *cut-IR* and overexpression of *Atg1*, *Atg5*, or *Atg8a* results in partial rescue of dendritic growth and branching deficits. (B-D) Quantitative analyses reveal that C-III Atg1 overexpression in a *cut-IR* loss-of-function background (CIII>Atg1;cut-IR) partially rescues reductions in the total number of dendritic branches (B) and number of terminal dendrites (D), but fails to rescue deficits in total dendritic length (C) relative to *cut-IR* loss-of-function C-III neurons (CIII>cut-IR), while Atg5 overexpression in a *cut-IR* loss-of-function background partially rescues reductions in the total number of dendritic branches (B) and total dendritic length (C) but fails to rescue reductions in the number of terminal dendrites (D), and Atg8a overexpression in a *cut-IR* background partially rescues all three morphological aspects. Statistics: one-way ANOVA with Dunnett’s multiple comparisons test (* = p≤0.05, *** = p≤0.001). For detailed genotypes see S1 Table. For detailed statistics see S2 Table.

Previous studies have demonstrated that *cut* mutant C-III md neurons exhibit defects in dendritic growth and loss of terminal dendritic filopodia-like branches, which are a characteristic feature of C-III neurons [25]. Consistent with these previous findings, we find that C-III-specific RNAi knockdown of *cut* leads to reductions in growth and a dramatic loss of dendritic terminal branches relative to WT controls (Fig 3A). If Cut utilizes the basal autophagy machinery to promote growth and dendritic terminal branching, then we would predict that upregulation of autophagic function may be sufficient to rescue aspects of defects in dendritic arborization associated with *cut* disruption. To test this hypothesis, we simultaneously knocked down *cut* and overexpressed the autophagy initiator kinase Atg1 in C-III neurons. Consistent with our prediction, Atg1 overexpression in a *cut-IR* loss-of-function genetic background resulted in a partial rescue of dendritic morphology defects relative to *cut-IR* alone (Fig 3A). Atg1 overexpression leads to significant increases in both the total number of dendritic branches (p<0.0001) and number of terminal dendrites (p = 0.0010), although total dendritic length is not rescued (p = 0.1882) (Fig 3B–3D). To further validate this rescue effect, we also performed comparative analyses with overexpression of Atg5 and Atg8a in a *cut-IR* background. Expression of these *Atg* genes resulted in partial rescue of the *cut-IR* phenotype (Fig 3A). Interestingly, these two genes, which function later in the autophagic process than Atg1, demonstrated significant rescue of dendritic length (Atg5: p = 0.0004, Atg8a: p = 0.0343) but less significant rescue of total dendritic branches (Atg5: p = 0.0124, Atg8a: p = 0.0172) and terminal dendrites (Atg5: not significant, Atg8a: p = 0.0396) relative to Atg1 (Fig 3B–3D). Collectively, these results support a role for the basal autophagy pathway as a downstream effector of Cut-mediated dendrite morphogenesis.

### Homeostatic regulation of basal autophagy is required for class-specific dendritic growth and branching

**Fig 4:**
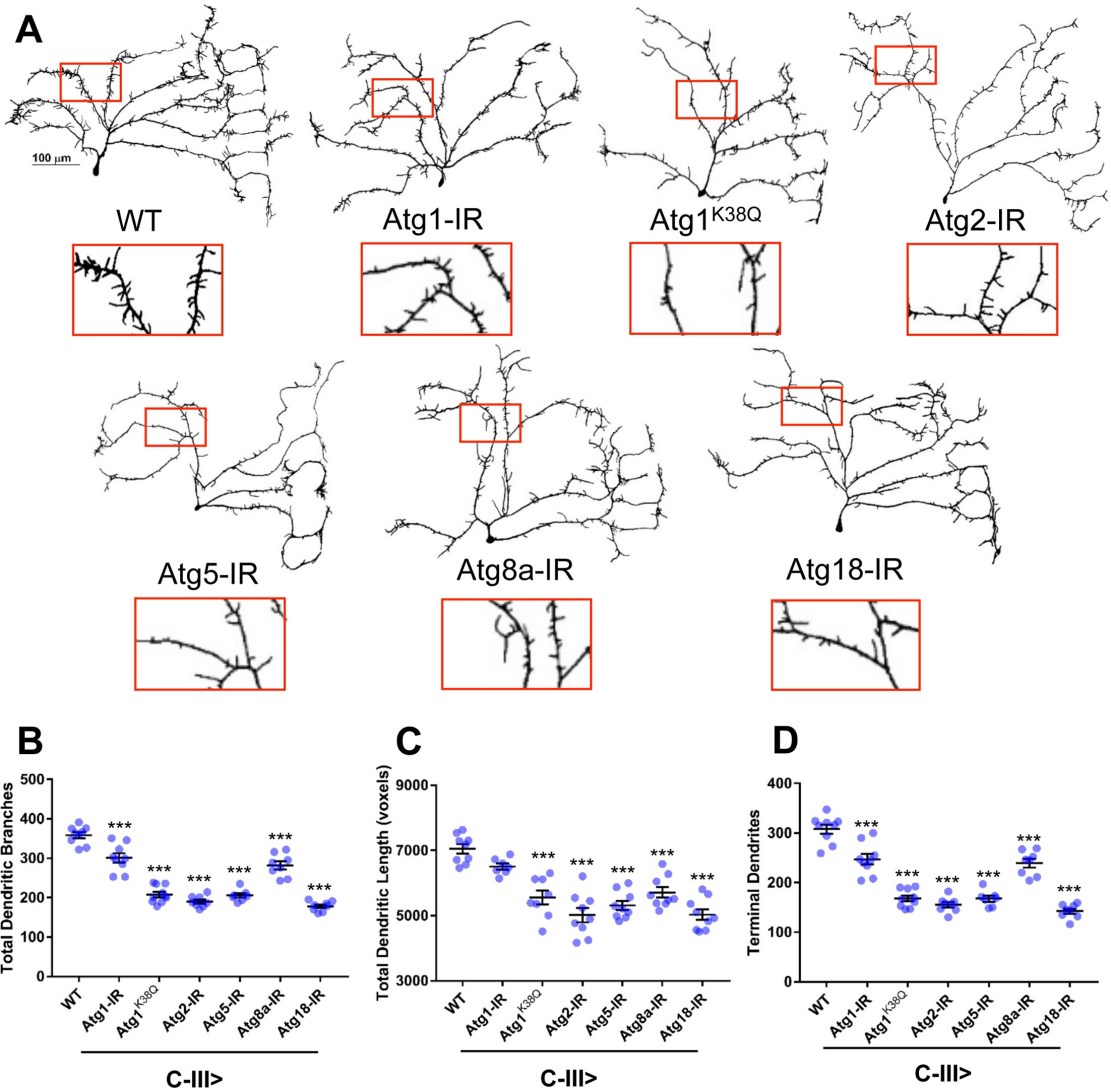
*Atg* genes are required to promote C-III md neuron dendritic growth and terminal branching. (A) Representative confocal images of third instar larval C-III ddaF md neurons. Compared to WT, knockdown of autophagic activity via an *Atg1* kinase dead transgene (K38Q) and *UAS-Atg-IR* transgenes results in reductions in dendritic growth and terminal branching. Insets present magnified view of corresponding dendritic regions between genotypes. (B-D) Quantitative analyses reveal reductions in the total number of dendritic branches (B), total dendritic length (C), and number of terminal dendrites (D) upon knockdown of *Atg* genes. Statistics: one-way ANOVA using Dunnett’s multiple comparisons test (* = p≤0.05, *** = p≤0.001). For detailed genotypes see S1 Table. For detailed statistics see S2 Table.

We next conducted phenotypic analyses to investigate the putative roles of autophagy in regulating cell type-specific dendritic development in C-III and C-IV md neurons, which natively express Cut at high and moderate levels, respectively. Relative to controls, the most notable effect of C-III specific knockdown of *Atg* genes was a reduction in the occurrence of terminal dendritic filopodia-like branches emanating from the primary arbors (Fig 4A). Quantitative morphometric analyses identified significant reductions in the total number of dendritic branches and number of terminal dendrites upon knockdown of all *Atg* genes analyzed, as well as significant reductions in all but *Atg1-IR* for total dendritic length. These results suggest that the major effect on branching was localized to C-III dendritic terminals. These morphological defects were likewise observed with C-III-specific expression of the *Atg1*^*K38Q*^ kinase dead transgene (Fig 4B–4D, see S2 Table for p-values).

**Fig 5:**
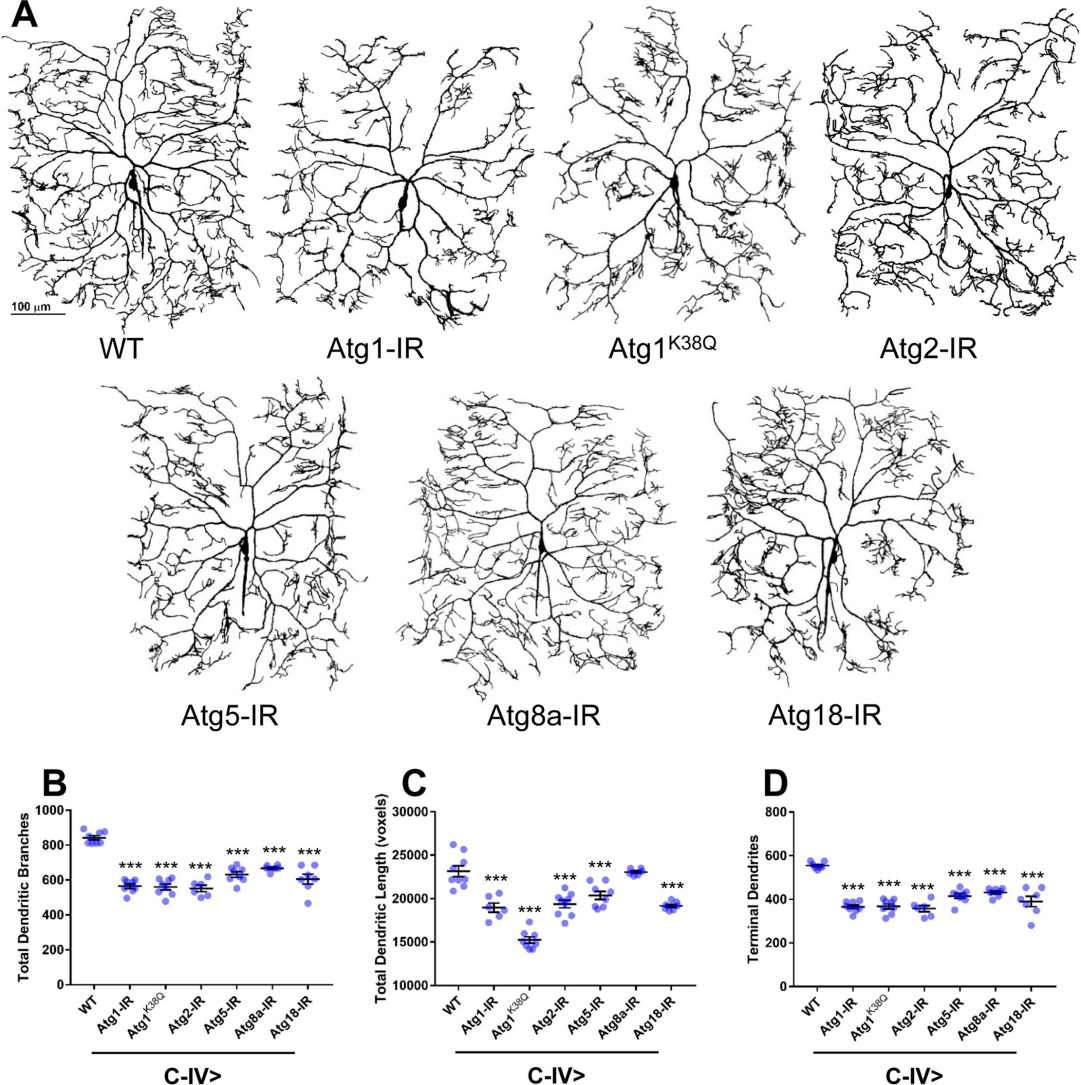
*Atg* genes function in regulating C-IV dendritic development and terminal branching. (A) Representative confocal images of third instar larval C-IV ddaC md neurons. Compared to WT, knockdown of autophagic activity via an *Atg1* kinase dead transgene (K38Q) and *UAS-Atg-IR* transgenes results in reductions in growth and terminal branching. **(**B-D) Quantitative analyses reveal reductions in the total number of dendritic branches (B) for all *Atg* genes investigated, decreases in total dendritic length (**C**) for all *Atg* genes investigated except *Atg8a*, and decreases in the number of terminal dendrites (D) without exception. Statistics: one-way ANOVA using Dunnett’s multiple comparisons test (*** = p≤0.001). For detailed genotypes see S1 Table. For detailed statistics see S2 Table.

In the case of C-IV neurons, knockdown of *Atg* genes resulted in variable qualitative phenotypic defects largely manifesting as reductions in dendritic arbor growth and terminal branching (Fig 5A). Quantitative analyses revealed significant reductions in the total number of dendritic branches (Fig 5B) and number of dendritic terminals (Fig 5D) for all *Atg* genes analyzed, whereas significant reductions total dendritic length (Fig 5C) were observed for all of the *Atg* genes except *Atg8a* (see S2 Table for p-values).

**Fig 6:**
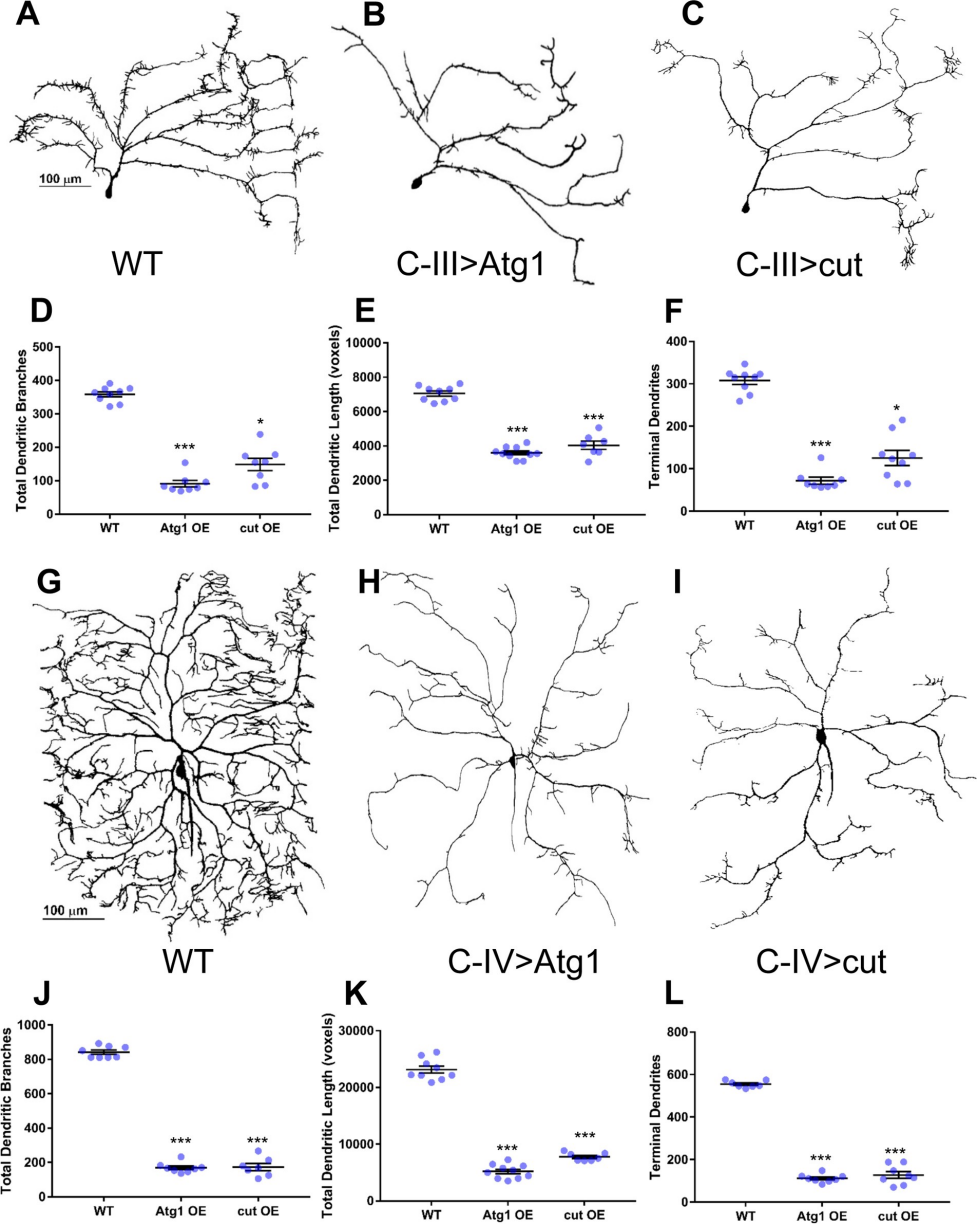
Atg1 or Cut overexpression restricts dendritic growth and higher order terminal branching. (A-C) Representative confocal images of third instar larval dorsal C-III ddaF md neurons. (G-I) Representative confocal images of third instar larval C-IV ddaC md neurons. Compared to WT (A,G), overexpression of Atg1 (B,H) or Cut (C,I) results in reductions in dendritic growth and terminal branching. Quantitative analyses reveal significant decreases in the total number of dendritic branches (D,J), total dendritic length (E,K), and number of terminal branches (F,L). Statistics: one-way ANOVA using Dunnett’s multiple comparisons test (E, J-L) or Kruskal-Wallis test using Dunn’s multiple comparisons test (D,F) (* = p≤0.05, *** = p≤0.001). For detailed genotypes see S1 Table. For detailed statistics see S2 Table. OE = overexpression.

Previous studies have implicated both insufficient and excessive autophagic induction in neurodegeneration as well as degeneration of axo-dendritic processes in patients suffering from a variety of neurodegenerative disorders including Alzheimer’s (AD), Parkinson’s (PD) and Huntington’s (HD) diseases (4,12,18). These findings suggest that homeostatic regulation of autophagy is important for maintaining neuritic architecture and neuronal survival. To investigate how excessive autophagic induction may impact md neuron dendritic development, we conducted gain-of-function phenotypic analyses of the Atg1 initiator kinase in C-III and C-IV neurons. Relative to controls, *Atg1* overexpression drastically decreased dendritic growth and higher order terminal branching in both C-III and C-IV md neurons (Fig 6A, 6B, 6G and 6H) resulting in severe reductions (p<0.0001 in all cases) in the number of dendritic branches (Fig 6D and 6J), total dendritic length (Fig 6E and 6K), and number of terminal dendrites (Fig 6F and 6L) in both C-III and C-IV md neurons. Interestingly, when we examine the effects of Cut overexpression in C-III and C-IV neurons, the phenotypic defects observed are strikingly similar to those observed with Atg1 overexpression in these neurons (Fig 6C and 6I). This observation is quantitatively supported in that Cut overexpression led to significant reductions in the total number of dendritic branches (Fig 6D and 6J**)**, total dendritic length (Fig 6E and 6K), and number of terminal dendrites (Fig 6F and 6L) that were consistent with the Atg1 overexpression phenotypes.

Collectively, these analyses suggest that the autophagy pathway is required to promote terminal dendritic branching in both C-III and C-IV md neurons as well as to regulate cell type-specific dendritic arborization architectures. Moreover, these loss-of-function studies are in line with findings from the suppression analyses in C-I neurons where knockdown of *Atg* genes appears to largely suppress the formation of Cut-induced dendritic growth and terminal filopodial branching (Fig 2). Furthermore, gain-of-function analyses reveal that excessive autophagic induction leads to severe reductions in dendritic growth and branching. Combined with the loss-of-function studies, this suggests that homeostatic regulation of the basal autophagy pathway is required for class-specific dendritic arborization.

### *Atg1* interacts with the DLK pathway in C-IV md neurons

**Fig 7:**
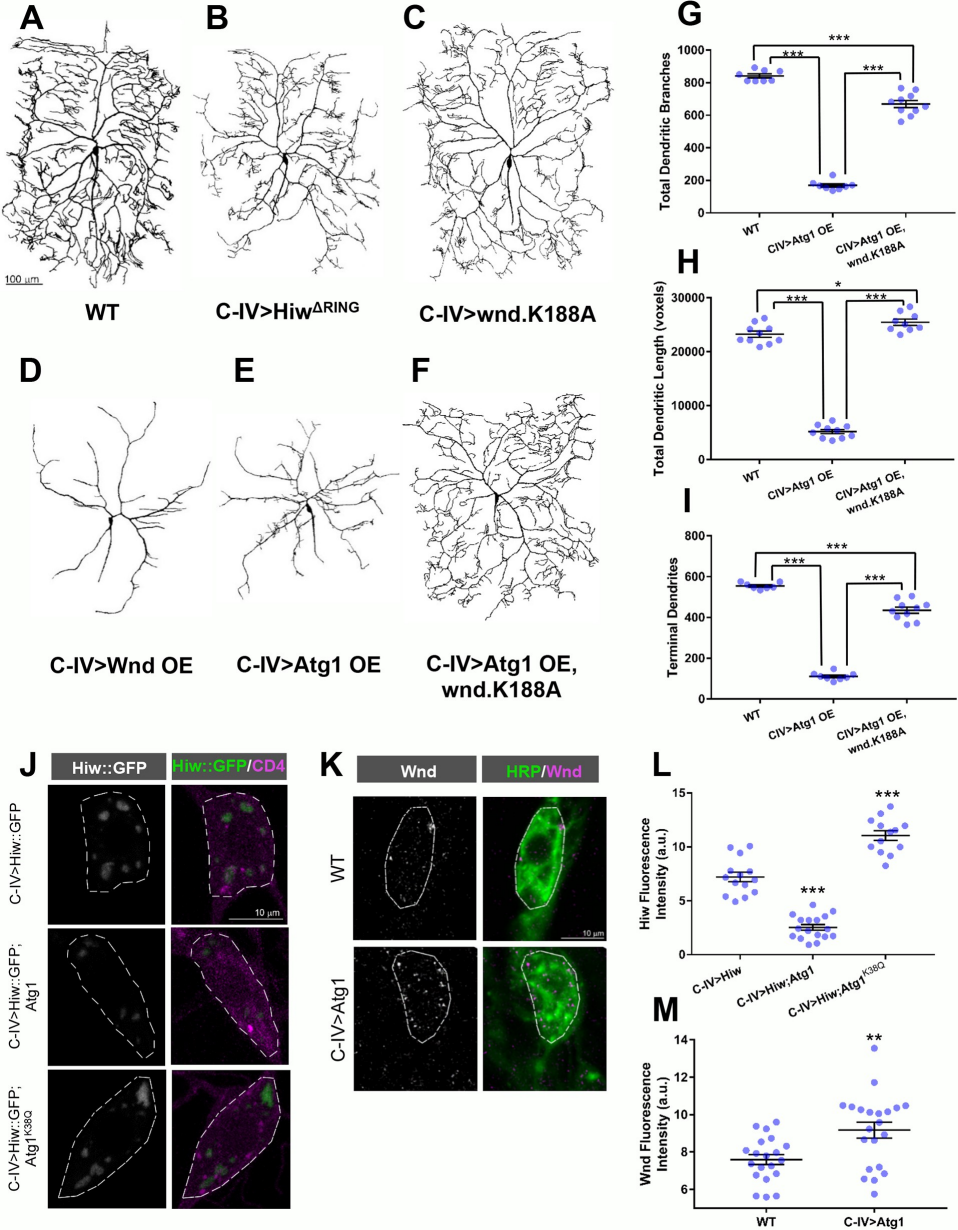
Atg1 negatively regulates Hiw and positively regulates Wnd in C-IV md neurons. (A-F) Representative confocal images of third instar larval C-IV ddaC md neurons. Compared to WT controls (A), disruption of the E3 ubiquitin ligase activity of *Hiw* (*Hiw*^*ΔRING*^) (B) and overexpression of the MAPKKK Wnd (D) result in reductions to C-IV dendritic growth and higher order terminal branching while overexpression of a kinase dead Wnd (C) has no notable effect on morphology, consistent with previous studies [45]. (E) Atg1 overexpressing C-IV neurons likewise exhibit defects in dendritic growth and higher order terminal branching consistent with defects observed with Wnd overexpression (D), and these defects are rescued when Atg1 overexpression is coupled with expression of kinase dead Wnd (F). (G-I) Quantitative analyses reveal significant decreases in the total number of dendritic branches, total dendritic length, and number of terminal branches for Atg1 overexpression as ^compared^ to WT. Co-expression of Atg1 and kinase dead Wnd (wnd.K188A), however, demonstrates significant rescue of all measured aspects of the Atg1 overexpression phenotype. (J) Representative confocal images of third instar larval C-IV md neurons expressing *UAS-Hiw*::*GFP* alone compared to C-IV md neurons expressing *UAS-Hiw*::*GFP* along with*UAS-Atg1* or *UAS-Atg1*^*K38Q*^. *ppk-hCD4*::*tdTomato* was used for C-IV md neuron visualization. (L) Quantification of GFP signal (fluorescence intensity) reveals a significant (p<0.0001) decrease in GFP-tagged Hiw expression upon Atg1 overexpression, whereas increased (p<0.0001) Hiw::GFP expression was observed upon expression of the kinase dead Atg1^K38Q^. (K) Representative confocal images of third instar larval WT C-IV md neurons and neurons overexpressing *UAS-Atg1* labeled with anti-Wnd antibodies. HRP labeling was used for neuron visualization. (M) Quantitative analysis reveals a significant (p = .0038) increase in Wnd expression (fluorescence intensity) with Atg1 overexpression. Statistics: one-way ANOVA using Dunnett’s multiple comparisons test (G-I, L) or two-tailed unpaired t-test with Welch’s correction (M) (** = p≤0.01, *** = p≤0.001). For detailed genotypes see S1 Table. For detailed statistics see S2 Table. OE = overexpression; a.u. = arbitrary units.

To gain insight into putative mechanisms by which the autophagy pathway may regulate cell type-specific md neuron dendrite morphogenesis, we focused on potential interactions with the dual leucine zipper kinase (DLK) pathway. Previous studies demonstrated that the autophagy pathway regulates synaptic and axonal growth via degradation of the E3 ubiquitin ligase Highwire (Hiw) in CNS neurons [15], and that disruptions of the DLK pathway, which is comprised of Hiw and the MAPKKK Wallenda (Wnd), produce bimodal and opposing effects on axonal and dendritic development of C-IV md neurons [45]. More precisely, Hiw functions to promote C-IV dendritic arbor growth and branching, whereas Wnd suppresses it [45]. Consistent with previous findings, we independently confirmed these phenotypic requirements in C-IV neurons by loss-of-function and gain-of-function studies of *hiw* and *wnd*, respectively. C-IV specific expression of a *hiw* mutant transgene (*UAS-Hiw*^*ΔRING*^) in which the Hiw E3 ubiquitin ligase RING domain is mutated generating a loss-of-function effect revealed defects in dendritic arbor growth and branching (Fig 7B) reminiscent of the defects observed with expression of the *Atg1*^*K38Q*^ kinase dead transgene (Fig 5A). In contrast, C-IV overexpression of Wnd dramatically reduced dendritic growth and higher order terminal branching (Fig 7D) in a manner that was phenotypically similar to the defects observed with Atg1 overexpression (Fig 7E). We therefore investigated whether the autophagy pathway may retain the same regulatory relationship with the DLK pathway in dendritic development as previously observed in axon growth and synapse development [15]. To assess this putative regulatory relationship we expressed a kinase dead Wnd transgene, *UAS-wnd*.*K188A*, in C-IV md neurons alone and in combination with Atg1 overexpression (Fig 7C and 7F). Consistent with previous findings [45], expression of the kinase dead Wnd transgene resulted in no notable morphological defects (Fig 7C), however co-expression with Atg1 resulted in dramatic and significant (p<0.0001 for all three measures) rescue of the morphological deficits induced by Atg1 overexpression (Fig 7F–7I), suggesting that Wnd functions downstream of Atg1 to regulate dendritic morphology in C-IV neurons. To assess the involvement of Hiw, we expressed a *UAS-Hiw*::*GFP* transgene [33] in C-IV md neurons with and without co-expression of *UAS-Atg1*. Live imaging of *Hiw*::*GFP* fluorescence revealed a significant (p<0.0001) decrease in the levels of GFP-tagged Hiw in neurons co-expressing *UAS-Atg1* relative to controls, thereby suggesting that Atg1 overexpression negatively regulates Hiw expression in C-IV neurons (Fig 7J and 7L). To further examine this relationship we co-expressed *UAS-Hiw*::*GFP* and the *Atg1*^*K38Q*^ kinase dead transgene. If Atg1 negatively regulates Hiw then we would expect to see an increase in *Hiw*::*GFP* fluorescence as the kinase dead Atg1 outcompetes native Atg1, and consistent with this prediction, we observed a significant (p<0.0001) increase in levels of GFP-tagged Hiw (Fig 7J and 7L). Based upon the negative regulatory relationship between Hiw and Wnd, we predicted that Atg1-mediated downregulation of Hiw would lead to a concomitant increase in Wnd expression levels. To investigate this, we compared Wnd expression levels in control and Atg1 overexpressing C-IV neurons and discovered a notable increase in punctate Wnd staining in the cell body (Fig 7K). Quantitative analysis revealed significant (p = 0.0038) increases in Wnd expression upon Atg1 overexpression relative to WT controls (Fig 7M). These results suggest that Atg1 can interact with the DLK pathway by regulating both Hiw and Wnd expression levels and that autophagy may utilize the DLK pathway to direct C-IV dendrite morphogenesis.

### Autophagic induction partially rescues dendritic defects under conditions of cellular stress induced by polyglutamine toxicity

**Fig 8:**
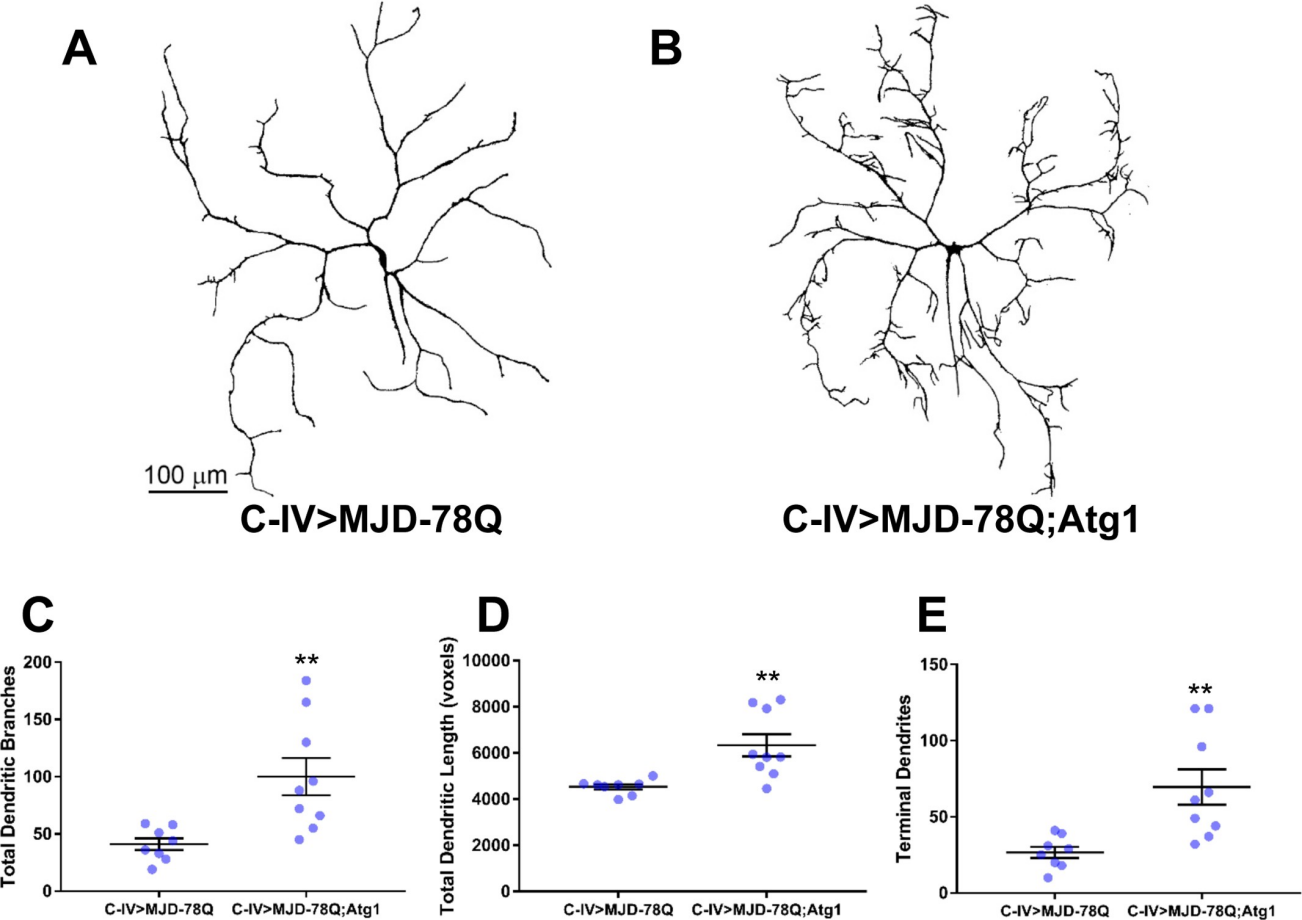
Atg1 overexpression partially rescues dendritic atrophy in a neuronal model of SCA3 polyQ toxicity. (A,B) Representative confocal images of third instar larval C-IV ddaC md neurons expressing *MJD-78Q* with and without co-expression of *Atg1*. Expression of *MJD-78Q* alone (A) results in severe dendritic atrophy. Simultaneous expression of *MJD-78Q* and *Atg1* (B) results in partial rescue of dendritic atrophy leading to increased growth and higher order branching. (C-E) Quantitative analyses reveal partial rescue of total number of dendritic branches (p = 0.0067) (C), total dendritic length (p = 0.0053) (D), and number of terminal dendrites (p = 0.0056) (E). Statistics: two-tailed unpaired t-test with Welch’s correction (** = p≤0.01). For detailed genotypes see S1 Table. For detailed statistics see S2 Table.

Autophagy has been demonstrated to play an essential neuroprotective role with respect to inhibiting neurodegeneration and loss of neuritic processes [4]. This led us to question whether autophagic induction may be capable of ameliorating loss of dendritic complexity associated with conditions of cellular stress. Recent studies in md neurons have modeled the neurodegenerative-like defects that occur in the polyglutamine (polyQ) disease spinocerebellar ataxia type 3 (SCA3), also known as Machado-Joseph disease (MJD). These studies revealed that expression of pathogenic SCA1 and SCA3 proteins results in severe dendritic atrophy [46]. Consistent with previous analyses, we found that C-IV overexpression of a transgene encoding a C-terminal truncated form of SCA3 (*UAS-MJD-78Q*) that retains a long polyQ repeat leads to severe defects in dendritic growth and higher order terminal branching presumptively linked to polyQ toxicity (Fig 8A). To determine whether the severe dendritic growth and branching deficits associated with SCA3 pathology could be rescued by autophagic induction, we overexpressed the Atg1 initiator kinase in C-IV neurons expressing the MJD-78Q transgene. Relative to MJD-78Q alone, Atg1 overexpression led to a partial rescue of the dendrite growth and higher order branching defects resulting from expression of SCA3 aggregates (Fig 8A and 8B). Quantitative morphometric analyses reveal significant rescue of the number of dendritic branches (Fig 8C, p = 0.0067), total dendritic length (Fig 8D, p = 0.0053), and number of terminal dendrites (Fig 8E, p = 0.0056). These results suggest that Atg1-mediated autophagic induction is capable of partially rescuing neurodegenerative-like dendritic arbor atrophy in the SCA3 aggregate toxicity model.

## Discussion

As a highly conserved lysosomal degradative pathway, autophagy has been shown to play a wide variety of mechanistic roles in regulating cellular homeostasis as well as in remodeling terminally differentiated cells [6–8,47–50]. Basal autophagy operates continuously as a catabolic housekeeping mechanism for turnover of misfolded proteins or cellular aggregates as well as recycling of damaged organelles, and can be acutely upregulated under conditions of cellular stress or injury. Postmitotic neurons are well known to require high levels of basal autophagy with defects in the process being linked to neurodegenerative cell death, aberrant synapse development, and axo-dendritic dystrophy [12,13,15,51–55]. Disruptions in *Atg* genes and autophagic function have been linked to the accumulation of ubiquitin-positive and other abnormal protein aggregates that contribute to pathological features of a variety of neurodegenerative disorders including PD and HD [4,17,19,45]. A hallmark of many neurodegenerative disease states is the degeneration of neuritic processes including axons, dendrites and synapses prior to neuronal cell death. However, less is known regarding the constitutive role of basal autophagy in regulating cell type-specific dendritic homeostasis or the mechanisms by which the autophagic pathway may be transcriptionally regulated in order to contribute to dendritic diversification and homeostasis.

Our results suggest that homeostatic regulation of the basal autophagy pathway is important in mediating aspects of cell type-specific dendritic arborization including terminal branching in *Drosophila* md sensory neurons. These findings are in contrast with a previous report that demonstrated a role of autophagy in the context of Nmnat-mediated protection against hypoxia-induced dendrite degeneration in C-IV md neurons, however did not identify any functional roles of basal autophagy for C-IV dendritic integrity [56]. Our work further suggests that the homeodomain transcription factor Cut regulates the expression of select *Atg* genes and that the autophagy pathway functions as a downstream effector of Cut-mediated cell type-specific dendritic development. Several lines of evidence support a positive regulatory role for Cut on components of the autophagy pathway: (1) ectopic expression of Cut in C-I md neurons reveals upregulated mRNA expression of *Atg* genes involved in autophagic induction, Atg protein cycling, and autophagosome vesicle completion; (2) Cut ectopic expression upregulates Atg8a protein expression, whereas Cut-specific knockdown in C-III and C-IV md neurons, which normally express Cut, leads to downregulation of Atg8a protein expression as well as downregulation of *Atg1*, *Atg2*, *Atg5*, *Atg8a*, and *Atg18* mRNA expression levels; (3) genetic suppressor analyses involving *Atg* gene knockdown in C-I md neurons ectopically expressing Cut support a role for basal autophagy as a downstream effector of Cut-mediated dendritic growth and terminal branching; and (4) overexpression of *Atg* genes (*Atg1*, *Atg5*, *Atg8a*) in a *cut* loss-of-function background can partially rescue defects in C-III md neuron dendritic growth and terminal branching. While these analyses are indicative of a positive regulatory relationship between Cut and components of the autophagic machinery, whether Cut directly or indirectly regulates these *Atg* genes remains to be determined. Our work demonstrates, however, that Cut is not absolutely required for Atg protein expression or autophagosome formation as Atg8a labeling of autophagosomes is observed in C-I md neurons that do not normally express detectable levels of Cut, suggesting that other transcriptional regulators likely also play important roles in directing *Atg* gene expression. The observations that disruption of individual *Atg* genes only partially suppresses Cut overexpression effects on C-I md neuron dendritic growth and filopodial-like terminal branching and that overexpression of Atg1 alone is insufficient to fully rescue *cut* loss-of-function dendritic defects in C-III md neurons can be explained in light of recent studies demonstrating that Cut functions via a variety of cellular processes in regulating dendritic development [3]. Recent neurogenomic studies demonstrate that Cut positively regulates the expression of hundreds of genes in md neurons [36] and functional studies have implicated molecules involved in cytoskeletal regulation [36,43,57,58], secretory pathway function [23,59], cell adhesion [39], and ribosomal regulatory function [36] as downstream effectors of Cut-mediated dendritic growth and branching, including the formation of dendritic filopodial-like terminal branches characteristic of C-III md neurons. These previous findings, coupled with our current results implicating autophagy, reveal that Cut exerts complex transcriptional effects on a broad spectrum of cellular processes that converge to promote dendritic diversity in md neurons and regulate terminal dendritic branching.

Insufficient or excessive levels of autophagic activity can lead to neuritic degeneration, highlighting the importance of homeostatic regulation of autophagy [16]. With respect to the constitutive role of basal autophagy in regulating dendritic morphogenesis under normal cellular conditions, our work suggests that homeostatic control of autophagy is required for cell type-specific dendritogenesis in C-III and C-IV md neurons. In both C-III and C-IV neurons, knockdown of *Atg* gene function leads to reductions in growth and terminal dendritic branching. In the case of C-III md neurons, this branching defect appears to manifest as a reduction in terminal branches, whereas in C-IV md neurons, we observed variable defects upon *Atg* gene knockdown leading to reductions in dendritic growth and terminal branching. Interestingly, C-IV expression of the kinase dead *Atg1*^*K38Q*^ transgene led to the most dramatic phenotypic effect with notable reductions in terminal arbor branching. Thus, insufficient autophagy appears to have major effects on cell type-specific dendritic terminal branching in C-III and C-IV neurons, whereas lower order dendritic branches do not appear to be affected. In contrast to lower order branches, studies have demonstrated that higher order terminal branches of md neurons are highly dynamic with respect to extension and retraction [60–62]. While the precise role of basal autophagy in regulating terminal dendritic branching is not yet clear, perhaps the relatively dynamic nature of these branches requires autophagy to support membrane or organelle turnover that may be required in branch extension and/or retraction, thereby contributing to cell type-specific arborization profiles.

A growing body of evidence indicates that excessive autophagy can also contribute to neurodegeneration and dystrophy of axo-dendritic processes, though the mechanism of action remains incompletely understood. In the case of neurodegenerative diseases such as AD, PD, and HD, degenerating neurites observed in brain tissue samples display increased levels of autophagy-related vesicular compartments [16] and in LRRK2 PD models, stimulating autophagy can exacerbate the dendritic retraction that may occur via excessive mitochondrial breakdown [63]. Consistent with these findings, our results reveal that Atg1 overexpression in C-III and C-IV md neurons leads to dramatic reductions in dendritic growth and terminal branching, suggesting that excessive autophagic induction contributes to dendritic atrophy. These findings are consistent with a previous report on the effects of Atg1 overxpression in C-IV neurons [56]. Interestingly, Cut overexpression in these neurons leads to highly similar phenotypic defects. The precise mechanisms by which Cut or Atg1 overexpression contribute to reductions in dendritic growth and branching remains unclear, however these results highlight the importance of homeostatic transcriptional and autophagic function in directing cell type-specific dendritogenesis. Moreover, the observation that Atg1 overexpression alone can induce these dendritic deficits suggests that autophagy induction is a critical step in the process and that other components of the autophagy pathway may not be rate limiting in C-III or C-IV md neurons.

How might the autophagy pathway mechanistically interact with other cell signaling pathways to regulate cell type-specific dendritic morphogenesis? A pair of intriguing studies have suggested that interaction with the DLK pathway may be one mechanism by which autophagy can exert control over this process [15,45]. In the *Drosophila* CNS, autophagy has been demonstrated to positively regulate the development of the larval neuromuscular junction (NMJ) [15]. Increased autophagy leads to an NMJ overgrowth phenotype that phenotypically mirrors effects seen in *hiw* E3 ubiquitin ligase mutants and this overgrowth phenotype can be suppressed by the MAPKKK protein Wnd. Autophagy interacts with the DLK pathway by negatively regulating Hiw expression in order to promote NMJ growth [15]. Interestingly, the roles of Hiw and Wnd are bimodal, contributing to compartmentalization in C-IV md neurons by producing opposing effects on axonal and dendritic development [45]. In axons Hiw restrains growth, whereas in dendrites it promotes C-IV arborization and Wnd suppresses dendritic growth. Our results demonstrate that similar to the regulatory relationship observed in the motor neuron NMJ, Atg1 can regulate the DLK pathway. Autophagic induction via Atg1 overexpression appears to downregulate the expression of Hiw and lead to a concomitant increase in the expression of Wnd in C-IV md neurons. Phenotypically, reduction of Hiw function impairs dendritic growth and branching, whereas overexpression of Wnd or Atg1 suppresses higher order dendritic terminal branching. As Atg1 overexpression leads to an upregulation of Wnd expression and produces phenotypically similar effects on dendritic arbor morphology which can be rescued by interfering with Wnd function, homeostatic control of autophagy induction may be important in regulating the relative expression levels of Hiw and Wnd to promote C-IV dendritic morphology.

Conditions of cellular stress can trigger an acute upregulation of basal autophagy as a mechanism to clear toxic protein aggregates or damaged organelles that may otherwise contribute to neurodegenerative states. Recent studies demonstrate that knockdown of autophagy via *Atg5* [64] as well as *Atg12* and *p62* [65] led to an increased accumulation of cytoplasmic SCA3 aggregates resulting in exacerbated retinal deterioration in *Drosophila* eye degeneration models. This suggests that autophagy may play a neuroprotective role by clearing pathogenic aggregates. Using a *Drosophila* model of SCA3 polyQ toxicity, we found that autophagic induction via Atg1 overexpression resulted in a partial rescue of neurodegenerative-like dendritic arbor atrophy defects. These findings are consistent with recent evidence demonstrating that upregulation of autophagy via overexpression of Beclin-1 (Atg6) enhances SCA3 aggregate clearance [66], and suggest that autophagy may protect against severe dendritic degeneration through the removal of these cytoplasmic aggregates.

In summary, these studies provide mechanistic insights into transcriptional regulation of basal autophagy and highlight the importance of homeostatic control of basal autophagy function in promoting cell type-specific dendritic arborization during normal development and under conditions of cellular stress.

## Supporting information

S1 Table Genotypes of larvae used in this study. (PDF)

S2 Table Statistical analyses used in this study. (PDF)
