# Supplementary material for: Basal autophagy is required for promoting dendritic terminal branching in Drosophila sensory neurons
Source: PLoS One. 2018 Nov 5;13(11):e0206743. doi: 10.1371/journal.pone.0206743 (PMC6218061; doi:10.1371/journal.pone.0206743)
Supplement: S1 Table — (PDF) [file pone.0206743.s001.pdf]

S1 Table: Genotypes of larvae used in this study

Figure 1A-C	w^{1118}; +; GAL4$^{2\text{-}21}$,UAS-mCD8::GFP/+<br>w^{1118}; UAS-cut/+; GAL4$^{2\text{-}21}$,UAS-mCD8::GFP/+
Figure 1D	w^{1118}; +; GAL4$^{2\text{-}21}$,UAS-mCD8::GFP (C-I)<br>w^{1118}; ppk-GAL4,UAS-mCD8::GFP,ppk-GAL80;+ (C-III)<br>w^{1118}; +; ppk1.9-GAL4,UAS-mCD8::GFP (C-IV)
Figure 1E	w^{1118}; GAL4^{477},UAS-mCD8::GFP/+; ppk1.9-GAL4,UAS-mCD8::GFP/+<br>$y^1sc^*v^1$; GAL4^{477},UAS-mCD8::GFP/+; ppk1.9-GAL4,UAS-mCD8::GFP/ UAS-cut$^{RNAi(HMS00924)}$
Figure 1F-H	w^{1118}; GAL4$^{21\text{-}7}$,UAS-mCD8::GFP/+;+<br>$y^1sc^*v^1$; GAL4$^{21\text{-}7}$,UAS-mCD8::GFP/+; UAS-cut$^{RNAi(HMS00924)}$/+
Figure 2	w^{1118}; +; GAL4$^{2\text{-}21}$,UAS-mCD8::GFP/+<br>w^{1118}; UAS-cut/+; GAL4$^{2\text{-}21}$,UAS-mCD8::GFP/+<br>y^1v^1; UAS-cut/+; GAL4$^{2\text{-}21}$,UAS-mCD8::GFP/UAS-Atg1$^{RNAi(JF02273)}$<br>y^1,w^{1118}; UAS-cut/+; GAL4$^{2\text{-}21}$,UAS-mCD8::GFP/UAS-Atg1^{K38Q}<br>$y^1sc^*v^1$; UAS-cut/+; GAL4$^{2\text{-}21}$,UAS-mCD8::GFP/UAS-Atg2$^{RNAi(HMS01198)}$<br>y^1v^1; UAS-cut/+; GAL4$^{2\text{-}21}$,UAS-mCD8::GFP/UAS-Atg5$^{RNAi(JF02703)}$<br>$y^1sc^*v^1$; UAS-cut/+; GAL4$^{2\text{-}21}$,UAS-mCD8::GFP/UAS-Atg8a$^{RNAi(HMS01328)}$<br>$y^1sc^*v^1$; UAS-cut/+; GAL4$^{2\text{-}21}$,UAS-mCD8::GFP/UAS-Atg18$^{RNAi(HMS01193)}$
Figure 3	w^{1118}; nompC-GAL4,UAS-mCD8::GFP/+; +<br>$y^1sc^*v^1$; nompC-GAL4,UAS-mCD8::GFP/+; UAS-cut$^{RNAi(HMS00924)}$/+<br>$y^1sc^*v^1$; nompC-GAL4,UAS-mCD8::GFP/+;UAS-cut$^{RNAi(HMS00924)}$/UAS-Atg1^{6B}<br>$y^1sc^*v^1$; nompC-GAL4,UAS-mCD8::GFP/+;UAS-cut$^{RNAi(HMS00924)}$/ UAS-eGFP-Atg5<br>$y^1sc^*v^1$; nompC-GAL4,UAS-mCD8::GFP/+;UAS-cut$^{RNAi(HMS00924)}$/ UAS-Atg8a.GFP
Figure 4	w^{1118}; +; GAL4$^{19\text{-}12}$,UAS-CD4::tdGFP/+<br>y^1v^1; +; GAL4$^{19\text{-}12}$,UAS-CD4::tdGFP/UAS-Atg1$^{RNAi(JF02273)}$<br>y^1,w^{1118}; +; GAL4$^{19\text{-}12}$,UAS-CD4::tdGFP/UAS-Atg1^{K38Q}<br>$y^1sc^*v^1$; +; GAL4$^{19\text{-}12}$,UAS-CD4::tdGFP/UAS-Atg2$^{RNAi(HMS01198)}$<br>y^1v^1; +; GAL4$^{19\text{-}12}$,UAS-CD4::tdGFP/UAS-Atg5$^{RNAi(JF02703)}$<br>$y^1sc^*v^1$; +; GAL4$^{19\text{-}12}$,UAS-CD4::tdGFP/UAS-Atg8a$^{RNAi(HMS01328)}$<br>$y^1sc^*v^1$; +; GAL4$^{19\text{-}12}$,UAS-CD4::tdGFP/UAS-Atg18$^{RNAi(HMS01193)}$
Figure 5	w^{1118}; GAL4^{477},UAS-mCD8::GFP/+; ppk1.9-GAL4,UAS-mCD8::GFP/+<br>y^1v^1; GAL4^{477},UAS-mCD8::GFP/+; ppk1.9-GAL4,UAS-mCD8::GFP/ UAS-Atg1$^{RNAi(JF02273)}$<br>y^1,w^{1118}; GAL4^{477},UAS-mCD8::GFP/+; ppk1.9-GAL4, UAS-mCD8::GFP/UAS-Atg1^{K38Q}<br>$y^1sc^*v^1$; GAL4^{477},UAS-mCD8::GFP/+; ppk1.9-GAL4,UAS-mCD8::GFP/

	UAS-Atg2$^{RNAi(HMS01198)}$
	y^1v^1*; GAL4*^{477}*,UAS-mCD8::GFP/+; ppk1.9-GAL4,UAS-mCD8::GFP/ UAS-Atg5*$^{RNAi(JF02703)}$
	$y^1sc^*v^1$*; GAL4*^{477}*,UAS-mCD8::GFP/+; ppk1.9-GAL4,UAS-mCD8::GFP/ UAS-Atg8a*$^{RNAi(HMS01328)}$
	$y^1sc^*v^1$*; GAL4*^{477}*,UAS-mCD8::GFP/+; ppk1.9-GAL4,UAS-mCD8::GFP/ UAS-Atg18*$^{RNAi(HMS01193)}$
Figure 6A-F	w^{1118}*; +; GAL4*$^{19\text{-}12}$*,UAS-CD4::tdGFP/+*
	w^{1118}*; +; GAL4*$^{19\text{-}12}$*,UAS-CD4::tdGFP/UAS-Atg1*^{6B}
	w^{1118}*; UAS-cut/+; GAL4*$^{19\text{-}12}$*,UAS-CD4::tdGFP/+*
Figure 6G-L	w^{1118}*; GAL4*^{477}*,UAS-mCD8::GFP/+; ppk1.9-GAL4,UAS-mCD8::GFP/+*
	w^{1118}*; GAL4*^{477}*,UAS-mCD8::GFP/+; ppk1.9-GAL4,UAS-mCD8::GFP/ UAS-Atg1*^{6B}
	w^{1118}*; GAL4*^{477}*,UAS-mCD8::GFP/UAS-cut; ppk1.9-GAL4, UAS-mCD8::GFP/+*
Figure 7A-I	w^{1118}*; GAL4*^{477}*,UAS-mCD8::GFP/+; ppk1.9-GAL4,UAS-mCD8::GFP/+*
	w^{1118}*; GAL4*^{477}*,UAS-mCD8::GFP/UAS-hiw*$^{\Delta RING}$*; ppk1.9-GAL4,UAS-mCD8::GFP/+*
	w^{1118}*; GAL4*^{477}*,UAS-mCD8::GFP/UAS-wnd.K188A; ppk1.9-GAL4, UAS-mCD8::GFP/+*
	w^{1118}*; GAL4*^{477}*,UAS-mCD8::GFP/UAS-wnd.C; ppk1.9-GAL4,UAS-mCD8::GFP/+*
	w^{1118}*; GAL4*^{477}*,UAS-mCD8::GFP/+; ppk1.9-GAL4,UAS-mCD8::GFP/ UAS-Atg1*^{6B}
	w^{1118}*; GAL4*^{477}*,UAS-mCD8::GFP/UAS-wnd.K188A; ppk1.9-GAL4, UAS-mCD8::GFP/UAS-Atg1*^{6B}
Figure 7J,L	w^{1118}*; GAL4*^{477}*/UAS-GFP-hiw; ppk-hCD4::tdTomato/+*
	w^{1118}*; GAL4*^{477}*/UAS-GFP-hiw; ppk-hCD4::tdTomato/UAS-Atg1*^{6B}
	w^{1118}*; GAL4*^{477}*/UAS-GFP-hiw; ppk-hCD4::tdTomato/ UAS-Atg1*^{K38Q}
Figure 7K,M	w^{1118}*; GAL4*^{477}*,UAS-mCD8::GFP/+; ppk1.9-GAL4,UAS-mCD8::GFP/+*
	w^{1118}*; GAL4*^{477}*,UAS-mCD8::GFP/+; ppk1.9-GAL4,UAS-mCD8::GFP/ UAS-Atg1*^{6B}
Figure 8	w^{1118}*; GAL4*^{477}*,UAS-mCD8::GFP/UAS-MJD-78Q; ppk1.9-GAL4, UAS-mCD8::GFP/+*
	w^{1118}*; GAL4*^{477}*,UAS-mCD8::GFP/UAS-MJD-78Q; ppk1.9-GAL4, UAS-mCD8::GFP/UAS-Atg1*^{6B}
