# Supplementary material for: Basal autophagy is required for promoting dendritic terminal branching in Drosophila sensory neurons
Source: PLoS One. 2018 Nov 5;13(11):e0206743. doi: 10.1371/journal.pone.0206743 (PMC6218061; doi:10.1371/journal.pone.0206743)
Supplement: S2 Table — (PDF) [file pone.0206743.s002.pdf]

S2 Table: Statistical analyses used in this study.

Figure/Label	Passed Shapiro-Wilk Normality?	Statistical tests used	p-value	N value (neurons)
**Fig. 1B**				
WT	Yes			15
CI>cut	Yes	Unpaired t-test with Welch's correction	<0.0001	15
**Fig. 1E**				(samples)
Atg1	Yes	One-way ANOVA with Dunnett's multiple comparisons test	<0.0001	4
Atg2	Yes	One-way ANOVA with Dunnett's multiple comparisons test	<0.0001	4
Atg5	Yes	One-way ANOVA with Dunnett's multiple comparisons test	<0.0001	4
Atg8a	Yes	One-way ANOVA with Dunnett's multiple comparisons test	<0.0001	4
Atg18	Yes	One-way ANOVA with Dunnett's multiple comparisons test	<0.0001	4
**Fig. 1H**				
C-I WT	Yes			20
C-I cut-IR	Yes	Unpaired t-test with Welch's correction	0.1203	18
C-III WT	Yes			18
C-III cut-IR	Yes	Unpaired t-test with Welch's correction	<0.0001	18
C-IV WT	Yes			21
C-IV cut-IR	Yes	Unpaired t-test with Welch's correction	0.0093	19
**Fig. 2B**				
WT	Yes	Kruskal-Wallis test with Dunn's multiple comparisons test	<0.0001	9
CI>cut	Yes			10
CI>cut+Atg1-IR	No	Kruskal-Wallis test with Dunn's multiple comparisons test	<0.0001	15
CI>cut+Atg1^{K38Q}	Yes	Kruskal-Wallis test with Dunn's multiple comparisons test	0.0149	11
CI>cut+Atg2-IR	Yes	Kruskal-Wallis test with Dunn's multiple comparisons test	<0.0001	15
CI>cut+Atg5-IR	Yes	Kruskal-Wallis test with Dunn's multiple comparisons test	0.0039	20
CI>cut+Atg8a-IR	Yes	Kruskal-Wallis test with Dunn's multiple comparisons test	0.0230	9
CI>cut+Atg18-IR	Yes	Kruskal-Wallis test with Dunn's multiple comparisons test	0.0118	14
**Fig. 2C**				
WT	Yes	Kruskal-Wallis test with Dunn's multiple comparisons test	<0.0001	11
CI>cut	Yes			10
CI>cut+Atg1-IR	Yes	Kruskal-Wallis test with Dunn's multiple comparisons test	0.0014	16
CI>cut+Atg1^{K38Q}	Yes	Kruskal-Wallis test with Dunn's multiple comparisons test	0.0011	12
CI>cut+Atg2-IR	No	Kruskal-Wallis test with Dunn's multiple comparisons test	0.0033	16
CI>cut+Atg5-IR	Yes	Kruskal-Wallis test with Dunn's multiple comparisons test	0.0057	19
CI>cut+Atg8a-IR	Yes	Kruskal-Wallis test with Dunn's multiple	0.1711	8

S2 Table: Statistical analyses used in this study.

		comparisons test		
CI>cut+Atg18-IR	Yes	Kruskal-Wallis test with Dunn's multiple comparisons test	0.1002	14
**Fig. 2D**				
WT	Yes	Kruskal-Wallis test with Dunn's multiple comparisons test	<0.0001	8
CI>cut	Yes			9
CI>cut+Atg1-IR	No	Kruskal-Wallis test with Dunn's multiple comparisons test	0.0003	16
CI>cut+$Atg1^{K38Q}$	Yes	Kruskal-Wallis test with Dunn's multiple comparisons test	0.0158	11
CI>cut+Atg2-IR	Yes	Kruskal-Wallis test with Dunn's multiple comparisons test	<0.0001	16
CI>cut+Atg5-IR	Yes	Kruskal-Wallis test with Dunn's multiple comparisons test	0.0058	19
CI>cut+Atg8a-IR	Yes	Kruskal-Wallis test with Dunn's multiple comparisons test	0.0567	9
CI>cut+Atg18-IR	Yes	Kruskal-Wallis test with Dunn's multiple comparisons test	0.0051	15
**Fig.3B**				
CIII>cut-IR	Yes			8
CIII>Atg1;cut-IR	Yes	One-way ANOVA with Dunnett's multiple comparisons test	<0.0001	10
CIII>Atg5;cut-IR	Yes	One-way ANOVA with Dunnett's multiple comparisons test	0.0124	10
CIII>Atg8a;cut-IR	Yes	One-way ANOVA with Dunnett's multiple comparisons test	0.0172	9
**Fig.3C**				
CIII>cut-IR	Yes			9
CIII>Atg1;cut-IR	Yes	One-way ANOVA with Dunnett's multiple comparisons test	0.4021	10
CIII>Atg5;cut-IR	Yes	One-way ANOVA with Dunnett's multiple comparisons test	0.0004	9
CIII>Atg8a;cut-IR	Yes	One-way ANOVA with Dunnett's multiple comparisons test	0.0343	9
**Fig.3D**				
CIII>cut-IR	Yes			9
CIII>Atg1;cut-IR	Yes	One-way ANOVA with Dunnett's multiple comparisons test	0.0002	10
CIII>Atg5;cut-IR	Yes	One-way ANOVA with Dunnett's multiple comparisons test	0.2256	10
CIII>Atg8a;cut-IR	Yes	One-way ANOVA with Dunnett's multiple comparisons test	0.0396	9
**Fig. 4B**				
WT	Yes			9
C-III>Atg1-IR	Yes	One-way ANOVA with Dunnett's multiple comparisons test	<0.0001	9
C-III>$Atg1^{K38Q}$	Yes	One-way ANOVA with Dunnett's multiple comparisons test	<0.0001	10
C-III>Atg2-IR	Yes	One-way ANOVA with Dunnett's multiple comparisons test	<0.0001	8
C-III>Atg5-IR	Yes	One-way ANOVA with Dunnett's multiple comparisons test	<0.0001	7

S2 Table: Statistical analyses used in this study.

C-III>Atg8a-IR	Yes	One-way ANOVA with Dunnett's multiple comparisons test	<0.0001	8
C-III>Atg18-IR	Yes	One-way ANOVA with Dunnett's multiple comparisons test	<0.0001	8
**Fig. 4C**				
WT	Yes			9
C-III>Atg1-IR	Yes	One-way ANOVA with Dunnett's multiple comparisons test	0.1478	7
C-III>Atg1^{K38Q}	Yes	One-way ANOVA with Dunnett's multiple comparisons test	<0.0001	8
C-III>Atg2-IR	Yes	One-way ANOVA with Dunnett's multiple comparisons test	<0.0001	9
C-III>Atg5-IR	Yes	One-way ANOVA with Dunnett's multiple comparisons test	<0.0001	9
C-III>Atg8a-IR	Yes	One-way ANOVA with Dunnett's multiple comparisons test	<0.0001	9
C-III>Atg18-IR	Yes	One-way ANOVA with Dunnett's multiple comparisons test	<0.0001	9
**Fig. 4D**				
WT	Yes			9
C-III>Atg1-IR	Yes	One-way ANOVA with Dunnett's multiple comparisons test	<0.0001	9
C-III>Atg1^{K38Q}	Yes	One-way ANOVA with Dunnett's multiple comparisons test	<0.0001	9
C-III>Atg2-IR	Yes	One-way ANOVA with Dunnett's multiple comparisons test	<0.0001	8
C-III>Atg5-IR	Yes	One-way ANOVA with Dunnett's multiple comparisons test	<0.0001	7
C-III>Atg8a-IR	Yes	One-way ANOVA with Dunnett's multiple comparisons test	<0.0001	8
C-III>Atg18-IR	Yes	One-way ANOVA with Dunnett's multiple comparisons test	<0.0001	8
**Fig. 5B**				
WT	Yes			8
C-IV>Atg1-IR	Yes	One-way ANOVA with Dunnett's multiple comparisons test	<0.0001	8
C-IV>Atg1^{K38Q}	Yes	One-way ANOVA with Dunnett's multiple comparisons test	<0.0001	8
C-IV>Atg2-IR	Yes	One-way ANOVA with Dunnett's multiple comparisons test	<0.0001	6
C-IV>Atg5-IR	Yes	One-way ANOVA with Dunnett's multiple comparisons test	<0.0001	8
C-IV>Atg8a-IR	Yes	One-way ANOVA with Dunnett's multiple comparisons test	<0.0001	6
C-IV>Atg18-IR	Yes	One-way ANOVA with Dunnett's multiple comparisons test	<0.0001	7
**Fig. 5C**				
WT	Yes			9
C-IV>Atg1-IR	Yes	One-way ANOVA with Dunnett's multiple comparisons test	<0.0001	6
C-IV>Atg1^{K38Q}	Yes	One-way ANOVA with Dunnett's multiple comparisons test	<0.0001	8
C-IV>Atg2-IR	Yes	One-way ANOVA with Dunnett's multiple	<0.0001	9

S2 Table: Statistical analyses used in this study.

		comparisons test		
C-IV>Atg5-IR	Yes	One-way ANOVA with Dunnett's multiple comparisons test	<0.0001	8
C-IV>Atg8a-IR	Yes	One-way ANOVA with Dunnett's multiple comparisons test	<0.0001	6
C-IV>Atg18-IR	Yes	One-way ANOVA with Dunnett's multiple comparisons test	<0.0001	7
**Fig. 5D**				
WT	Yes			7
C-IV>Atg1-IR	Yes	One-way ANOVA with Dunnett's multiple comparisons test	<0.0001	8
C-IV>Atg1^{K38Q}	Yes	One-way ANOVA with Dunnett's multiple comparisons test	<0.0001	8
C-IV>Atg2-IR	Yes	One-way ANOVA with Dunnett's multiple comparisons test	<0.0001	6
C-IV>Atg5-IR	Yes	One-way ANOVA with Dunnett's multiple comparisons test	<0.0001	8
C-IV>Atg8a-IR	Yes	One-way ANOVA with Dunnett's multiple comparisons test	<0.0001	7
C-IV>Atg18-IR	Yes	One-way ANOVA with Dunnett's multiple comparisons test	<0.0001	7
**Fig. 6D**				
WT	Yes			9
Atg1 OE	No	Kruskal-Wallis test with Dunn's multiple comparisons test	<0.0001	8
Cut OE	Yes	Kruskal-Wallis test with Dunn's multiple comparisons test	0.0166	8
**Fig. 6E**				
WT	Yes			9
Atg1 OE	Yes	One-way ANOVA with Dunnett's multiple comparisons test	<0.0001	10
Cut OE	Yes	One-way ANOVA with Dunnett's multiple comparisons test	<0.0001	7
**Fig. 6F**				
WT	Yes			9
Atg1 OE	No	Kruskal-Wallis test with Dunn's multiple comparisons test	<0.0001	8
Cut OE	Yes	Kruskal-Wallis test with Dunn's multiple comparisons test	0.0106	9
**Fig. 6J**				
WT	Yes			8
Atg1 OE	Yes	One-way ANOVA with Dunnett's multiple comparisons test	<0.0001	8
Cut OE	Yes	One-way ANOVA with Dunnett's multiple comparisons test	<0.0001	7
**Fig. 6K**				
WT	Yes			9
Atg1 OE	Yes	One-way ANOVA with Dunnett's multiple comparisons test	<0.0001	10
Cut OE	Yes	One-way ANOVA with Dunnett's multiple comparisons test	<0.0001	7
**Fig. 6L**				
WT	Yes			7

S2 Table: Statistical analyses used in this study.

Atg1 OE	Yes	One-way ANOVA with Dunnett's multiple comparisons test	<0.0001	8
Cut OE	Yes	One-way ANOVA with Dunnett's multiple comparisons test	<0.0001	8
**Fig. 7G**				
WT				8
C-IV>Atg1 OE	Yes	One-way ANOVA with Dunnett's multiple comparisons test	<0.0001	8
C-IV>Atg1 OE,wnd.K188A	Yes	One-way ANOVA with Dunnett's multiple comparisons test	<0.0001 (vs.WT) <0.0001 (vs.Atg1 OE)	10
**Fig. 7H**				
WT				10
C-IV>Atg1 OE	Yes	One-way ANOVA with Dunnett's multiple comparisons test	<0.0001	10
C-IV>Atg1 OE,wnd.K188A	Yes	One-way ANOVA with Dunnett's multiple comparisons test	0.0154 (vs.WT) <0.0001 (vs.Atg1 OE)	9
**Fig. 7I**				
WT				7
C-IV>Atg1 OE	Yes	One-way ANOVA with Dunnett's multiple comparisons test	<0.0001	8
C-IV>Atg1 OE,wnd.K188A	Yes	One-way ANOVA with Dunnett's multiple comparisons test	<0.0001 (vs.WT) <0.0001 (vs.Atg1 OE)	10
**Fig. 7L**				
C-IV>Hiw	Yes			14
C-IV>Hiw;Atg1	Yes	One-way ANOVA with Dunnett's multiple comparisons test	<0.0001	17
C-IV>Hiw;Atg1^{K38Q}	Yes	One-way ANOVA with Dunnett's multiple comparisons test	<0.0001	13
**Fig. 7M**				
WT	Yes			20
C-IV>Atg1	Yes	Unpaired t-test with Welch's correction	0.0038	21
**Fig. 8C**				
C-IV>MJD-78Q	Yes			8
C-IV>MJD-78Q;Atg1	Yes	Unpaired t-test with Welch's correction	0.0067	9
**Fig. 8D**				
C-IV>MJD-78Q	Yes			8
C-IV>MJD-78Q;Atg1	Yes	Unpaired t-test with Welch's correction	0.0053	9
**Fig. 8E**				
C-IV>MJD-78Q	Yes			8
C-IV>MJD-78Q;Atg1	Yes	Unpaired t-test with Welch's correction	0.0056	9
